# Polymer Gel-Based Triboelectric Nanogenerators: Conductivity and Morphology Engineering for Advanced Sensing Applications

**DOI:** 10.3390/gels11090737

**Published:** 2025-09-13

**Authors:** Sabuj Chandra Sutradhar, Nipa Banik, Mohammad Mizanur Rahman Khan, Jae-Ho Jeong

**Affiliations:** 1 Department of Energy Materials Science and Engineering, Konkuk University, Chungju-si 27478, Republic of Korea; chandra@kku.ac.kr (S.C.S.); nipa1987@kku.ac.kr (N.B.); 2 Research Center for Green Energy Systems, Department of Mechanical Engineering, Gachon University, Seongnam-si 13120, Republic of Korea; 3 School of Mechanical Engineering, College of Engineering, Chung-Ang University, 84 Heukseok-ro, Dongjak-gu, Seoul 06974, Republic of Korea

**Keywords:** triboelectric nanogenerators (TENGs), polymer gels, self-powered sensors, conductivity enhancement, flexible electronics, wearable sensing platforms

## Abstract

Polymer gel-based triboelectric nanogenerators (TENGs) have emerged as versatile platforms for self-powered sensing due to their inherent softness, stretchability, and tunable conductivity. This review comprehensively explores the roles of polymer gels in TENG architecture, including their function as triboelectric layers, electrodes, and conductive matrices. We analyze four operational modes—vertical contact-separation, lateral-sliding, single-electrode, and freestanding configurations—alongside key performance metrics. Recent studies have reported output voltages of up to 545 V, short-circuit currents of 48.7 μA, and power densities exceeding 120 mW/m^2^, demonstrating the high efficiency of gel-based TENGs. Gel materials are classified by network structure (single-, double-, and multi-network), matrix composition (hydrogels, aerogels, and ionic gels), and dielectric medium. Strategies to enhance conductivity using ionic salts, conductive polymers, and nanomaterials are discussed in relation to triboelectric output and sensing sensitivity. Morphological features such as surface roughness, porosity, and micro/nano-patterning are examined for their impact on charge generation. Application-focused sections detail the integration of gel-based TENGs in health monitoring (e.g., sweat, glucose, respiratory, and tremor sensing), environmental sensing (e.g., humidity, fire, marine, and gas detection), and tactile interfaces (e.g., e-skin and wearable electronics). Finally, we address current challenges, including mechanical durability, dehydration, and system integration, and outline future directions involving self-healing gels, hybrid architectures, and AI-assisted sensing. This review expands the subject area by synthesizing recent advances and offering a strategic roadmap for developing intelligent, sustainable, and multifunctional TENG-based sensing technologies.

## 1. Introduction

The rapid advancement of wearable electronics, biomedical diagnostics, and environmental monitoring technologies has created an urgent demand for flexible, lightweight, and self-powered sensing systems [1,2,3,4,5,6,7,8]. Conventional sensors, which rely on external power sources such as batteries or capacitors, face limitations in terms of bulkiness, finite energy capacity, and environmental sustainability [9]. These constraints hinder the development of autonomous and long-lasting sensing platforms, particularly in dynamic and mobile applications. In response, TENGs have emerged as a promising solution, capable of converting ambient mechanical energy into electrical signals through contact electrification and electrostatic induction. TENGs have emerged as a promising technology for harvesting ambient mechanical energy, converting it into electrical signals through contact electrification and electrostatic induction [10,11,12,13,14,15,16,17,18].

Since their introduction in 2012 [19], TENGs have gained significant attention due to their high voltage output, simple fabrication, and compatibility with diverse materials [20,21,22]. Their working principle involves the generation of surface charges when two materials with differing electron affinities come into contact and separate. This mechanism is particularly effective at the nanoscale, where enhanced surface interactions amplify charge generation. Conventional triboelectric materials—for example, polyterafluoroethylene (PTFE), polyvinylidinefluoride (PVDF), and silicone elastomers—are often constrained by mechanical rigidity, limited biocompatibility, and poor conformity to irregular or dynamic surfaces [23,24,25,26].

To overcome these limitations, recent studies have explored the incorporation of polymer gels—such as hydrogels, ionogels, and organogels—into TENG architectures, offering improved flexibility, biocompatibility, and adaptability to complex surfaces [4,27,28,29,30,31,32,33]. Gel-based materials uniquely combine softness, stretchability, tunable conductivity, and biocompatibility, positioning them as ideal candidates for the development of next-generation flexible and wearable triboelectric nanogenerators [34]. Gels consist of three-dimensional polymer networks swollen with aqueous or ionic media, and can be tailored through various synthesis approaches, including physical or chemical crosslinking, solvent exchange, and the incorporation of conductive fillers such as carbon nanotubes, graphene, and MXenes [18,26,34].

The incorporation of gels into TENGs addresses several critical challenges [35,36,37,38]. Their mechanical compliance allows for conformal contact with irregular surfaces, enhancing triboelectric interaction and charge transfer [39]. Their hydrated or ionic nature facilitates ionic and electronic conductivity, enabling efficient signal transduction and energy harvesting. Moreover, many gel systems exhibit self-healing properties, which are essential for maintaining device integrity under repeated mechanical stress [39].

Recent investigations have demonstrated the effectiveness of gel-based TENGs across a wide range of applications, highlighting their potential in flexible and multifunctional sensing systems. Hydrogels have been used to fabricate stretchable and transparent TENGs capable of detecting subtle tactile inputs and powering wearable electronics [40,41,42]. Ionogels, with their stable ionic conductivity and low volatility, have shown promise in high-temperature and humidity-resistant sensing platforms [43,44,45,46,47]. Composite gels, incorporating nanomaterials and conductive polymers, have further enhanced triboelectric output and mechanical robustness [48,49,50]. These innovations have enabled the development of self-powered sensors for monitoring physiological signals (e.g., respiration, glucose levels, and tremors), environmental parameters (e.g., humidity, gas concentration, and fire detection), and human–machine interactions (e.g., electronic skin and gesture recognition) [17,20].

Despite notable progress, several challenges persist. In particular, the environmental stability of hydrogels remains a concern, as their tendency to dehydrate significantly compromises long-term performance [24]. Signal consistency under dynamic mechanical conditions and seamless integration with electronic and data-processing systems also require further optimization. Moreover, scalability and cost-effectiveness are critical for commercial translation [34].

Emerging trends in the field offer promising solutions. The development of self-healing gels can significantly enhance device longevity and reliability [51,52,53,54]. Hybrid systems, combining triboelectric and piezoelectric mechanisms, can broaden the sensing range and improve energy conversion efficiency [42]. The integration of artificial intelligence (AI) and machine learning algorithms with gel-based TENGs can enable real-time data interpretation, adaptive sensing, and predictive diagnostics [17].

This review presents an in-depth analysis of triboelectric nanogenerators incorporating polymer gels, emphasizing material design, operational principles, performance metrics, and application domains. We begin by discussing the fundamental roles of gels in TENG architecture, including their function as triboelectric layers, electrodes, and conductive matrices. Various operational modes—vertical contact-separation, lateral-sliding, single-electrode, and freestanding triboelectric-layer configurations—are examined with energy conversion mechanisms. Key performance indicators such as output voltage, current, and power density are analyzed alongside material properties and structural configurations.

Subsequent sections delve into the classification of gel materials based on network structure and matrix composition, highlighting hydrogels, aerogels, and ionic gels. Strategies for enhancing conductivity through ionic/electronic additives, nanomaterials, and hybrid composites are critically evaluated. The impact of surface morphology, including roughness, porosity, and micro/nano-patterning, on triboelectric output is also discussed.

Application-focused sections explore the integration of gel-based TENGs in health monitoring, environmental sensing, and tactile interfaces, with emphasis on wearable and intelligent systems. Finally, we address current challenges and outline future directions involving self-healing gels, multifunctional hybrid architectures, and AI-assisted sensing platforms. By synthesizing recent advancements and identifying strategic directions, this review aims to support the design and advancement of efficient, durable, and adaptable TENG-based sensing technologies for next-generation smart electronics.

## 2. Fundamentals of Polymer Gel-Based Triboelectric Nanogenerators

Polymer gel-based TENGs represent a cutting-edge approach to mechanical energy harvesting, particularly suited for flexible, wearable, and biocompatible electronics. These systems integrate the triboelectric effect and electrostatic induction with the unique properties of polymer gels, such as mechanical tunability, ionic conductivity, and environmental adaptability [12].

### 2.1. Functional Roles of Gels in TENG Architecture

Gel-based materials have emerged as multifunctional components in the design of TENGs, offering a unique combination of mechanical compliance, electrical tunability, and chemical adaptability [12,28]. Their soft, deformable nature and ability to host both ionic and electronic conductive pathways make them ideal for integration into flexible, wearable, and self-powered sensing systems [51,53]. Depending on their composition and structural configuration, gels can serve in several critical roles within TENG architectures, each contributing to the overall energy conversion efficiency and sensing performance.

#### 2.1.1. Triboelectric Layer

Gels can act as the active triboelectric surface, where contact electrification occurs during mechanical interaction. Their softness and viscoelasticity allow for enhanced conformal contact with counter surfaces, increasing the effective contact area and, consequently, the triboelectric charge density [55]. For instance, hydrogel-based triboelectric layers have demonstrated superior performance in wearable sensors due to their ability to adapt to skin curvature and motion [56,57]. Additionally, surface engineering techniques—such as micro-patterning or nano structuring—can be applied to gel surfaces to further enhance charge generation by increasing surface roughness and frictional interaction [58].

#### 2.1.2. Electrode Material

Conductive gels, particularly those embedded with nanomaterials like MXenes, carbon nanotubes (CNTs), graphene, or conductive polymers, can function as flexible electrodes within TENGs [33,59]. These gels form percolated conductive networks that facilitate efficient charge collection and transport while maintaining mechanical flexibility. Unlike rigid metal electrodes, gel-based electrodes can stretch, bend, and conform to dynamic surfaces without delamination or performance degradation [29]. This makes them especially suitable for skin-mounted electronics, implantable devices, and soft robotics.

#### 2.1.3. Electrolyte Medium

In hybrid TENG configurations, gels can serve as ionic conductors, enabling dual-mode energy harvesting that combines triboelectric and electrostatic induction mechanisms [12]. These gels typically contain mobile ions that redistribute under mechanical stimulation, enhancing the electrostatic potential difference between electrodes. This role is particularly important in liquid–solid TENGs and triboionic systems, where the gel acts as both a dielectric and an electrolyte [60]. The ionic conductivity of such gels can be tuned by adjusting ion concentration, adjusting pH, or incorporating ion-selective membranes.

#### 2.1.4. Structural Support

Beyond their electrical functions, gels contribute significantly to the mechanical integrity of TENG devices. Their elastic and viscoelastic properties allow them to absorb mechanical stress, recover from deformation, and maintain device stability under repeated loading cycles [17]. This structural role is crucial in applications involving wearable electronics, prosthetics, and soft robotic skins, where the device must endure continuous bending, stretching, and compression. Moreover, certain gels inherently possess self-healing and adhesive characteristics, which contribute to the long-term functionality and practical usability of TENG systems [55,61].

The multifunctionality of gel-based materials enables their integration into multiple layers of TENG architecture, from the triboelectric interface to the electrode and electrolyte components. Their tunable conductivity, adaptable morphology, and mechanical resilience make gel-based materials highly suitable for advanced sensing applications, including physiological monitoring, environmental detection, and human–machine interaction.

#### 2.1.5. Triboelectric Effect and Electrostatic Induction

The triboelectric effect, which reinforces the operation of TENGs, involves the generation of surface charges through contact and separation between two materials with differing electron affinities. In gel-based systems, materials such as hydrogels, organogels, and aerogels are employed either as triboelectric layers or electrodes. These gels are frequently modified with polar functional groups to enhance surface charge density and retention capabilities, thereby improving their triboelectric performance [62]. Once the triboelectric charges are generated, electrostatic induction facilitates the movement of electrons through an external circuit, thereby producing electrical energy. Notably, ionic-conductive gels, particularly hydrogels, support ion migration in addition to electron flow, which enhances the overall charge transport and energy conversion efficiency [63].

### 2.2. Working Principles and Structural Configurations of Gel-Based TENGs

TENGs operate through the synergistic coupling of contact-induced charge transfer and subsequent electrostatic potential generation [12]. When two materials with differing electron affinities come into contact and are subsequently separated, electrons transfer between their surfaces, creating a charge imbalance. This separation generates a potential difference that initiates electron flow through an external circuit, resulting in electrical output [55].

In gel-based TENGs, soft and deformable polymer gels—often with ionic conductivity—serve as one or both of the triboelectric layers. These gels offer enhanced mechanical compliance, biocompatibility, and surface adaptability, making them particularly suitable for flexible, wearable, and implantable energy harvesting systems [17].

**Figure 1:**
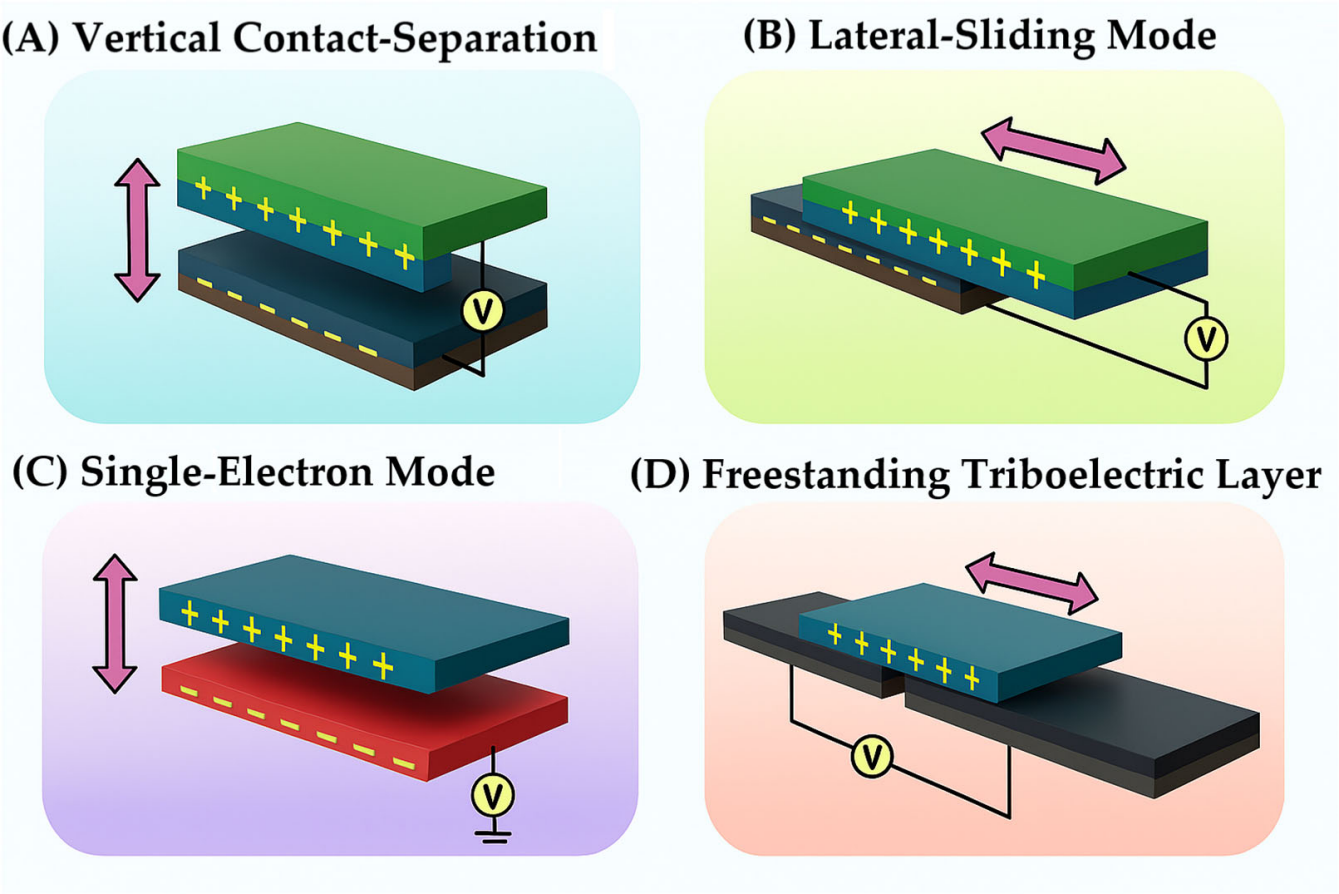
Schematic representation of the working principles and operational modes of TENGs: (**A**) vertical contact-separation; (**B**) single-electrode; (**C**) lateral-sliding; and (**D**) freestanding triboelectric-layer configurations.

To accommodate diverse application scenarios, TENGs have been developed in four primary working modes, each with distinct structural configurations and charge transfer dynamics: (i) vertical contact-separation (VCS) mode, (ii) lateral-sliding mode, (iii) single-electrode (SE) mode, and (iv) freestanding triboelectric-layer (FSTL) mode (Figure 1).

Gel-based TENGs have been successfully implemented across these modes, with the choice of configuration tailored to the specific mechanical input and application requirements [4,64,65].

#### 2.2.1. Vertical Contact-Separation (VCS) Mode

The VCS mode is among the most extensively explored and foundational mechanisms in TENG research [66,67]. This mode operates through the periodic contact and separation of two triboelectric layers—typically composed of materials with differing electron affinities—thereby generating alternating current (AC) via contact electrification and electrostatic induction (Figure 1A). Upon the application of mechanical force, the TENG device brings the two layers—typically a gel-based triboelectric material and a dielectric film—into contact. The contact between the layers facilitates electron transfer, leading to the generation of opposite surface charges. When the mechanical force is released, the layers separate vertically, inducing a potential difference between the electrodes due to the spatial displacement of the triboelectric charges. This potential deviation drives electrons through an external circuit from one electrode to the other, generating an electric current. The resulting potential difference drives electron flow through an external circuit, thereby generating an electric current between the electrodes. As the layers return to their original position and re-contact, the potential difference reverses, causing the flow of electrons in the opposite direction. This cyclic deformation produces a continuous AC output, as illustrated in Figure 1A [19,68]. Importantly, one of the triboelectric layers is typically non-conductive, ensuring that the generated charges remain localized on the surface rather than dissipating through conduction [19]. These immobile surface charges are essential for sustaining the electrostatic field that drives current through the external load during each cycle of contact and separation.

In gel-based TENGs, the use of soft, stretchable hydrogels or ion-conductive gels enhances the conformal contact between layers, thereby increasing the effective contact area and improving charge generation [40].

The VCS mode is particularly suitable for applications involving pressure sensing, tactile feedback, and biomechanical energy harvesting, where vertical mechanical stimuli are prevalent [69,70,71]. Its simplicity in design and high energy conversion efficiency make it a foundational mode for both fundamental research and the practical deployment of gel-based TENGs.

#### 2.2.2. Lateral-Sliding Mode

In TENGs operating in lateral-sliding mode, two key frictional interactions—normal contact and in-plane sliding—are essential for effective charge generation. As illustrated in Figure 1B, the TENG structure facilitates charge accumulation through lateral displacement between two triboelectric surfaces [72]. This sliding motion induces triboelectrification, which arises from frictional forces and leads to periodic mechanical deformation at the interface. Such deformation results in a potential difference across the surfaces, driving the flow of charge carriers through an external circuit. At equilibrium, the polymeric layers are in intimate contact, maximizing the overlap and minimizing charge separation. The triboelectric effect causes one surface to accumulate positive charges, while the other becomes negatively charged, maintaining overall charge neutrality. One of the triboelectric layers typically acts as an insulator, ensuring that the surface charges remain stable over extended periods without significant leakage. During the sliding process, particularly when the positively charged top layer moves outward, the contact area decreases, initiating in-plane charge separation. This dynamic separation induces an electric field aligned with the sliding direction, elevating the potential at the top electrode. The magnitude of the generated voltage is directly influenced by the extent of sliding and the triboelectric properties of the materials involved [73,74,75].

The lateral-sliding mode of TENGs has been successfully employed in a range of practical applications, including energy harvesting from table drawers, gesture recognition through five-finger motion sensors, and low-cost paper-based page-mark sensors. These implementations demonstrate the versatility of lateral-sliding TENGs in capturing mechanical energy from everyday movements and converting it into usable electrical signals for self-powered sensing systems [76,77,78].

#### 2.2.3. Single-Electrode (SE) Mode

SE mode represents a simplified and highly adaptable configuration of TENGs, particularly suitable for wearable, touch-sensitive, and mobile applications [72]. Unlike conventional TENG architectures that require two interconnected electrodes, the SE mode utilizes a single active electrode connected to ground, while the opposing triboelectric surface is provided by an external object—such as a human finger, textile, or environmental surface—that interacts with the device through contact or sliding.

As illustrated in Figure 1C, the SE-mode TENG operates based on the synergistic effects of contact electrification and electrostatic induction. When a mechanical stimulus—such as tapping, sliding, or pressing—is applied, the triboelectric layer (often a gel-based material) comes into contact with the external surface, generating opposite surface charges due to triboelectrification. Upon separation, the displaced charges create a potential difference between the electrode and ground, which drives electron flow through the external circuit and generates an AC signal [19].

This mode is particularly advantageous in scenarios where electrode interconnection is impractical, such as fingertip-driven interfaces, touchscreens, floor-based energy harvesting, and mobile object tracking. For example Wang et al. introduces a gel polymer electrode-based single-electrode SE-TENG integrated into a flexible touch sensor, enabling real-time detection of finger motion and pressure without requiring a second electrode [79].

The SE mode also supports minimalistic and compact device designs, making it ideal for integration into smart textiles, skin-mounted sensors, and implantable biomedical devices. Gel materials—such as ionic hydrogels, conductive polymer gels, and cellulose-based composites—are ideal for SE-mode TENGs due to their softness, biocompatibility, and adaptability to dynamic surfaces.

The single-electrode mode of TENGs has been effectively integrated into various advanced sensing platforms, including soft robotics, wearable kneepad sensors, and electronic skin (e-skin) systems [76,77,78]. These applications leverage the mode’s structural simplicity and adaptability to conformal surfaces, enabling efficient energy harvesting and self-powered sensing in dynamic environments.

#### 2.2.4. Freestanding Triboelectric-Layer (FSTL) Mode

The FSTL mode operates similarly to the single-electrode configuration, wherein mechanical disturbances from freely moving objects are harnessed to generate electrical energy. As illustrated in Figure 1D, the FSTL mode involves a mobile triboelectric layer that interacts with a pair of interconnected electrodes, typically arranged to ensure full coverage by the freestanding component [72].

Upon contact between the freestanding layer and the electrodes, electrostatic induction facilitates the flow of charge carriers across the system. This interaction can occur through planar or rotational sliding, depending on the design of the electrodes and the motion of the freestanding layer [80]. In some configurations, the laterally aligned electrodes are replaced with vertically arranged ones, allowing the conductive freestanding layer to move between them. To prevent charge leakage in such cases, a dielectric layer is often introduced beneath the electrodes [81]. Notably, the FSTL mode can also function in a non-contact regime. Once the triboelectric surfaces are initially charged through contact, subsequent sliding is not strictly necessary. Even with sufficient separation, the freestanding layer can continue to induce charge transfer as it moves over the electrodes. This approach significantly reduces surface wear, thereby extending the operational lifetime of the device [82].

The freestanding mode of TENGs has demonstrated promising utility in diverse domains, including energy harvesting from dynamic sports activities and environmental remediation, such as the removal of organic pollutants [82,83,84]. These applications leverage the mode’s ability to convert continuous lateral motion into electrical energy, enabling self-powered systems for both wearable technologies and sustainable environmental solutions.

### 2.3. Key Performance Metrics of Polymer Gel-Based Triboelectric Nanogenerators

The performance of polymer gel-based TENGs is evaluated through a set of standardized metrics that reflect their energy conversion efficiency and practical applicability. These metrics include output voltage, output current, power density, and charge density, each influenced by the material composition, structural design, and environmental conditions of the device [85].

#### 2.3.1. Output Voltage

Output voltage in gel-based TENGs typically ranges from 50 to 500 volts, depending on the triboelectric properties of the gel and the configuration of the device. Hydrogels and organogels, when engineered with high surface polarity and optimized contact interfaces, can achieve voltages at the upper end of this spectrum [86,87,88].

#### 2.3.2. Output Current and Charge Density

Output current varies from microamperes to milliamperes, largely influenced by the ionic conductivity and effective contact area of the gel. The incorporation of conductive fillers such as MXenes, graphene, or carbon nanotubes into the gel matrix has been shown to improve charge mobility and increase current output. In a recent study, Guo et al. [89] demonstrated that a stretched poly(vinylidene fluoride-co-trifluoroethylene) (PVDF-TrFE)) film, engineered for enhanced permittivity and breakdown strength, achieved record values of 2.8 mC/m^2^ in charge density and 6.2 × 10^5^ J/m^3^ in output energy density. This advancement highlights the critical role of dielectric optimization in enhancing TENG performance.

#### 2.3.3. Power Density

Power density, a critical metric for evaluating the energy harvesting capability of TENGs, has seen substantial improvement in recent designs. Values exceeding 100 W/m^2^ have been reported, particularly in systems utilizing nanocomposite-enhanced gels. These composites leverage the synergistic effects of polymer matrices and nanofillers to boost triboelectric performance.

#### 2.3.4. Application Relevance and Integration

These innovations have enabled gel-based TENGs to power a range of self-sustained devices, including motion trackers, environmental monitors, and wearable health sensors. Their integration into the Internet of Things (IoT) ecosystem is becoming increasingly viable, as they offer lightweight, flexible, and biocompatible solutions for energy autonomy [90]. The development of self-driven charge excitation systems further expands the application potential of TENGs by enabling direct power delivery to multiple electronic components without external energy sources [91,92].

Overall, the continuous refinement of performance metrics and material engineering strategies is driving the evolution of polymer gel-based TENGs from laboratory prototypes to practical energy solutions. Future research is expected to focus on scalable fabrication, long-term durability, and multifunctional integration to meet the growing demands of wearable and autonomous electronic systems.

## 3. Polymer Gels in TENGs: Material Properties and Conductivity

### 3.1. Classification and Properties of Gel Materials for TENG Applications

The intrinsic composition of gel materials governs their physical and electrical properties, which in turn determine their suitability for TENG applications. Typically, gel materials consist of three core components: a network structure, a matrix material, and a contained liquid [92,93]. While the network structure is generally a three-dimensional crosslinked polymer network, variations in the matrix and liquid components lead to diverse performance characteristics across different gel types.

#### 3.1.1. Classification Based on Network Structure

**Scheme 1:**
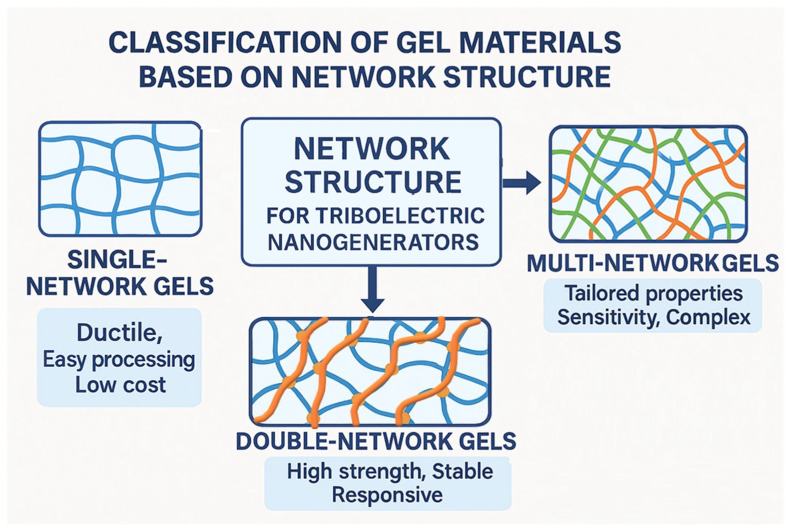
Schematic classification of gel materials based on network structure.

Gel materials used in TENGs can be classified based on the number of polymer networks they contain. These include single-network, double-network, and multi-network gels, each offering distinct structural and functional advantages (Scheme 1) [93,94,95].

##### Single-Network Gels

Single-network gels consist of a single crosslinked polymer network, formed through chemical or physical interactions. These gels are typically ductile, easy to process, and cost-effective, making them suitable for scalable TENG applications [96,97,98]. For example, Jing et al. [99] developed a core/shell gel fiber using photocrosslinking within a transparent silica gel hollow fiber, serving both as a protective electrode layer and friction layer in a graphene/silk-based TENG (GS-TENG). This design enhanced stretchability and avoided issues like metal electrode cracking and liquid leakage. Similarly, Parida et al. [100] used a PVA-ionic liquid hydrogel electrode with high transparency (~92%) and excellent stretchability (~700%). However, single-network gels often suffer from limited mechanical strength and long-term stability, which can lead to performance degradation. As a result, their use in TENGs is typically limited to applications requiring high flexibility and low cost.

##### Double-Network (DN) Gels

DN gels are composed of two interpenetrating polymer systems, typically combining a rigid and a ductile phase. This structure significantly enhances mechanical strength, stability, and responsiveness [25,48,49,50]. Zhao et al. [101] developed a multifunctional DN hydrogel-based TENG (DHN-TENG), achieving a short-circuit current of 16.2 μA, a transferred charge of 97.3 nC, and an open-circuit voltage of 270.5 V. The device exhibited excellent stretchability (up to 566%) and was used in a sensor array for pressure and temperature mapping. Further, Zhang et al. [102] designed a DN conductive hydrogel with freeze resistance and flexibility, which was utilized in a gel-polymer triboelectric nanogenerator (GP-TENG) that delivered an open-circuit voltage of 182 V and a short-circuit current of 2.61 μA at 25 °C. It demonstrated high sensitivity (0.336 kPa^−1^) and a low detection limit (0.016 kPa), maintaining performance even at −18 °C. Hsu et al. [22] developed a polypropylene-reinforced triboelectric nanogenerator (PRP-TENG) capable of harvesting natural energy (sound, rain, and wind) to power greenhouses, increasing crop growth rates by 55–72%. The hydrogel also served as an ammonia sensor, capable of detecting concentrations ranging from 8.68 to 5000 ppm.

##### Multi-Network Gels

Multi-network gels incorporate more than two crosslinked polymer networks, offering even greater mechanical and functional versatility [24,103]. These networks may be chemically or physically crosslinked and can be composed of similar or different polymers. Compared to DN gels, multi-network gels provide enhanced multifunctionality in TENGs, including higher sensitivity and mechanical adaptability [104]. Wang et al. [105] introduced covalent and ionic crosslinking into a carboxymethyl chitosan/allyl glycidyl ether hydrogel (CCH), enhancing ionic conductivity and anti-freezing properties down to −40 °C. The CCH-based TENG maintained stable and reversible sensitivity to finger bending at subzero temperatures, making it suitable for low-temperature flexible electronics. However, multi-network gels also present challenges such as complex design, higher production costs, and integration difficulties in multifunctional systems. Table 1 presents the core material compositions along with the corresponding output voltage and current values for gel-based TENGs categorized by network structure [20].

#### 3.1.2. Classification by Matrix Composition

**Scheme 2:**
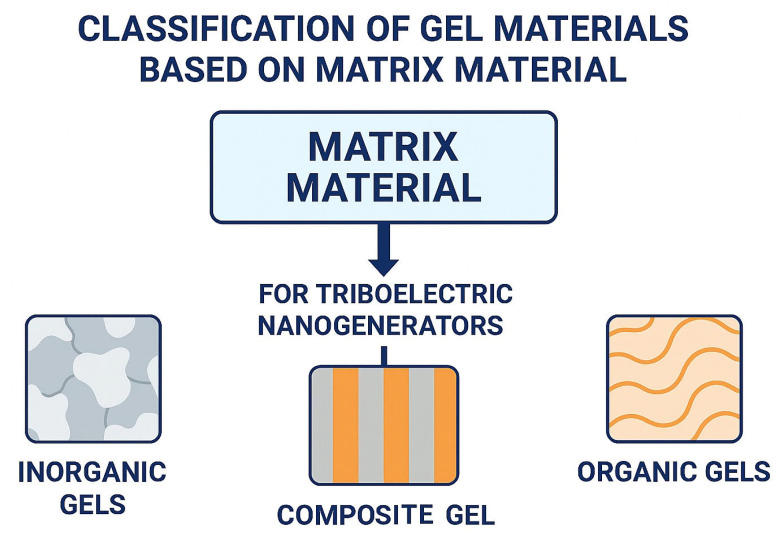
Schematic classification of gel materials based on matrix materials.

Gel materials are classified into inorganic, organic, and composite types, depending on the nature of their matrix components (Scheme 2) [108].

However, this classification is not always mutually exclusive. In some cases, researchers modify the matrix using various treatments to enhance specific properties, making it difficult to assign a gel to a single category. Therefore, a comprehensive and integrated approach is necessary when classifying gels based on matrix composition.

##### Inorganic Gels

Inorganic gels are primarily composed of silicates, metal oxides, or their composites, depending on the specific inorganic compounds used [108,109,110]. Silicate gels are known for their chemical stability, making them suitable for high-temperature, high-humidity, and corrosive environments [111,112,113]. For instance, Rezaei et al. [114] modified silica aerogels by grafting vinyltrimethoxysilane (VTMS) into polymethylhydrosiloxane (PMHS), resulting in a superhydrophobic surface characterized by a 162.31° water contact angle and thermal stability up to 518 °C. When used as the negative friction layer in a TENG, the modified aerogel achieved an output voltage of ~8 V, a 370% improvement over unmodified silica aerogels. This work significantly enhanced both hydrophobicity and energy harvesting capability in harsh environments.

Despite these advantages, silicate-based TENGs often suffer from poor electrical conductivity and limited friction performance, leading to relatively low output. In contrast, metal oxide gels address these limitations by offering better conductivity and triboelectric performance [115]. Zhou et al. [116] developed a zinc oxide nanofluid hydrogel (AP-Zn) exhibiting remarkable properties, including a tensile strain of 2750%, conductivity of 1.37 S/m, anti-freezing capability down to −20 °C, and self-healing ability. The resulting AP-Zn-TENG achieved an open-circuit voltage of ~135 V and could detect pressures as low as 0.025 kPa, including subtle vibrations from vocal cords, making it highly promising for self-powered biosensors.

Although promising, the number of studies on TENGs based on inorganic gels—especially composite gels combining silicates and metal oxides—remains limited. This is largely due to challenges such as poor flexibility, low mechanical strength, and complex fabrication processes, which hinder their application in wearable and flexible electronics. To enhance the applicability of inorganic gels in next-generation energy harvesting systems, future research should prioritize improving their flexibility and integration

##### Organic Gels

Organic gels are primarily made of organic macromolecular polymers, which may be natural (e.g., gelatin, sodium alginate, and chitosan), synthetic (e.g., polyacrylamide and polyvinyl alcohol), or composites of both [117,118,119]. Natural organic gels are formed through physical or chemical crosslinking of biomolecules like polysaccharides and proteins. These gels offer excellent biocompatibility and biodegradability, making them ideal for wearable biomedical TENGs [117,118,119]. For instance, Kim et al. [120] fabricated hyaluronic acid (HA) hydrogel membranes that were non-cytotoxic and promoted cell proliferation. When used as a friction layer in a TENG, the HA membrane generated an open-circuit voltage of ~20 V during contact with PTFE, demonstrating potential for implantable and biodegradable energy devices.

Despite these advantages, natural organic gels face limitations such as low triboelectric output, high preparation cost, poor mechanical strength, and limited durability. These shortcomings can be addressed by using synthetic organic gels, which offer enhanced performance [121,122]. Park et al. [123] modified polyvinyl chloride (PVC) gels with oxalic acid plasticizers of varying alkyl chain lengths. The DOA-modified PVC gel showed improved permittivity and conductivity, producing up to 198 V at 65 °C when used as a friction layer in a TENG, making it suitable for high-temperature self-powered systems.

Synthetic organogels also exhibit superior mechanical strength. Wang et al. [124] developed an electronic conductive hydrogel (CHS) for electrodes exhibiting 0.25 MPa tensile strength, 1015% fracture strain, and 1.22 MJ/m^3^ toughness. The term ‘conductive’ in this context refers to electronic conductivity, which is relevant to the electrode layer in TENGs, facilitating efficient electron transport and charge collection. The CHS-based TENG could detect subtle muscle movements like breathing and speaking, showing promise for biomonitoring and health management. The CHS-based TENG demonstrated sensitivity to subtle muscle activities such as breathing and speaking, highlighting its potential for biomonitoring and health management. To further enhance performance, composite organogels integrate natural and synthetic components, offering biocompatibility, mechanical robustness, and triboelectric efficiency. Zhang et al. [125] introduced an alkali-polyphenol autocatalytic system to synthesize lignosulfonate-based polyacrylamide (LSPAM) hydrogels. These hydrogels exhibited self-adhesive and self-healing properties due to abundant hydrogen bonding. When used in lignosulfonate-based polyacrylamide-based TENG (LS-TENGs), they achieved an open-circuit voltage of 265 V and a short-circuit current of 2.7 μA, offering a green, efficient, and low-cost solution for flexible electronics.

While organic gels offer many benefits, they also present challenges:

- Low mechanical strength can lead to deformation or rupture during operation.
- Poor environmental stability, especially sensitivity to humidity, affects performance.
- Low electrical conductivity limits energy conversion efficiency, though doping with conductive materials (e.g., poly(3,4-ethylenedioxythiophene:polystyrenesulfonate), PEDOT:PSS) can help, at the cost of increased complexity and expense.

Future improvements in organogels should focus on enhancing mechanical durability, environmental resilience, and electrical performance to meet the demands of next-generation TENG applications.

##### Composite Gels

Composite gels are formed by integrating two or more distinct material types—typically organic and inorganic components—to achieve enhanced mechanical, electrical, optical, and environmental properties compared to single-component gels [126,127]. These gels exhibit superior mechanical strength and ductility, often achieved by incorporating inorganic nanoparticles. Kim et al. [128] developed a catechol–chitosan–diatom hydrogel (CCDHG) for a wearable TENG (CCDHG-TENG), designed as an “M-type” tremor sensor. The term “M-type” refers to the M-shaped configuration of the Kapton film used in the sensor design, which contributes to enhanced mechanical adaptability and signal resolution. It achieved an open-circuit voltage of 110 V and a short-circuit current of 3.8 μA, enabling real-time monitoring of Parkinson’s disease symptoms using machine learning. Additionally, Bagchi et al. [129] developed a hydrogel-based TENG encapsulated in an Ecoflex layer, capable of withstanding mechanical forces up to 45 N and maintaining tensile properties of 233%. The device delivered a power density of 1.68 W/m^2^ and ~26% energy conversion efficiency, along with rapid self-healing and long-term stability. Composite gels also benefit from enhanced conductivity through the inclusion of conductive materials like carbon nanotubes or polymers. Xiao et al. [130] doped graphitic carbon nitride (g-C_3_N_4_) into a PVA hydrogel, producing a flexible and cost-effective electrode for TENGs. The resulting device reached a peak-to-peak open-circuit voltage of 80 V, significantly outperforming the pristine PVA hydrogel. The device delivered a power density of 1.68 W/m^2^ and ~26% energy conversion efficiency, along with rapid self-healing and long-term stability. Specifically, the dehydrated g-C_3_N_4_/PVA hydrogel demonstrated recovery of up to 80% of its original performance. Furthermore, a self-contact electrification (SCE) aerogel was engineered by incorporating a polyimide (SF-PI) containing fluoro and sulfone groups onto a graphene aerogel (GA) [131]. This structure enabled charge redistribution during deformation, generating electricity through internal contact electrification. The SCE-TENG could distinguish materials based on contact-induced voltage differences, paving the way for soft robotics and intelligent self-powered sensors.

Composite gels combine the strengths of both organic and inorganic gels:

- Inorganic gels offer mechanical robustness and environmental stability but lack conductivity and self-healing.
- Organic gels provide better conductivity and healing but suffer from lower mechanical strength and stability.
- Composite gels integrate these advantages, offering flexibility, durability, and enhanced triboelectric performance.

However, challenges remain in optimizing the mechanical properties, conductive stability, and environmental resilience. Continued research and innovation are essential to improve the performance and market viability of composite gels in TENG applications.

The primary material compositions, along with the corresponding output voltage and current values, are summarized in Table 2 for the gel-based TENGs categorized by matrix material.

#### 3.1.3. Classification Based on Medium Material

**Scheme 3:**
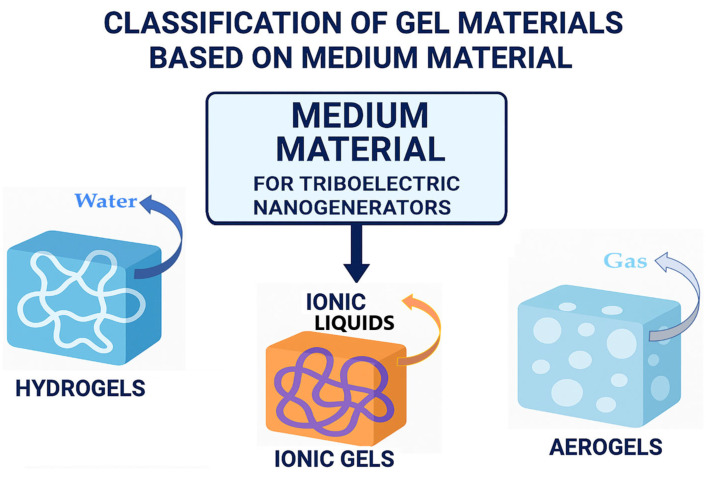
Schematic classification of gel materials based on medium materials.

Gel materials used in TENGs can be broadly categorized into hydrogels, aerogels, and ionic gels based on the type of medium they contain (Scheme 3). These three classifications encompass the majority of gel systems applied in triboelectric nanogenerators. A systematic classification approach provides clearer insight into the current development and application landscape of gel-based TENGs.

##### Hydrogels

Hydrogels are three-dimensional networks of hydrophilic polymer chains, primarily composed of water and water-soluble polymers such as PVA, PAM, and gelatin [79,132,133]. Hydrogels, as water-rich polymeric networks, have emerged as a foundational material in the development of flexible and biocompatible TENGs. Their intrinsic ionic conductivity, derived from the mobility of solvated ions (e.g., Na^+^, K^+^, and Cl^−^) within the hydrated matrix, plays a pivotal role in enhancing triboelectric performance. This conductivity is not only tunable through the manipulation of ion concentration and polymer composition but also synergistically interacts with the mechanical properties of the gel to facilitate efficient charge transfer during contact electrification. The ionic conductivity of hydrogels can be tuned by adjusting the ion concentration, polymer composition, and crosslinking density. Their high water content and flexibility make them suitable for biomedical, environmental, and wearable applications. Typical approaches to improve the ionic conductivity of gel matrices include incorporating conductive fillers or dopants [134,135,136], introducing free ions [137,138], and embedding conductive polymers [139,140,141].

The morphological characteristics of hydrogels—namely their softness, stretchability, and porous architecture—enable intimate contact with dynamic surfaces such as human skin, thereby improving the tribo [36] electric interface. These features are particularly advantageous for wearable and implantable sensing applications. Moreover, the incorporation of functional nanomaterials such as MXenes, graphene oxide, and carbon nanotubes into hydrogel matrices has been shown to significantly enhance both mechanical robustness and electrical conductivity, enabling multifunctional sensing capabilities [59,142,143,144]. Luo et al. [59] developed a flexible multifunctional hydrogel-based triboelectric nanogenerator (MH-TENG) by doping MXene nanosheets into PVA hydrogel. The device achieved an open-circuit voltage of 230 V in single-electrode mode. Building on this, Lai et al. [145] modified MXene (two-dimensional transition metal carbides/nitrides) surfaces with CPTMS (3-chloropropyltrimethoxysilane) and FOTS (1H,1H,2H,2H-perfluorooctyltriethoxysilane), enhancing the streaming vibration potential (SVP) and achieving an open-circuit voltage of 212 V. Similarly, Zhang et al. [146] reported a polymer-triboelectric surface-modified triboelectric nanogenerator (PTSM-TENG) incorporating tannic acid, sodium alginate, and MXene. Encapsulated in silicone rubber, the device reached a power density of 54.24 mW/m^2^ and was used in a glove-based human–machine interface system with 98.7% classification accuracy. Additionally, Wang et al. [147] enhanced PAM hydrogels with black phosphorus nanosheets, creating a photothermal-responsive hybrid triboelectric nanogenerator (HY-TENG). The device could decrypt temperature-sensitive patterns using infrared light, enabling secure information transmission. Gelatin-based hydrogels also show promise. Yan et al. [148] created an antibacterial hydrogel using fish gelatin (FG-Ag) and In situ nanoparticle generation. The FG-Ag TENG exhibited 2600% stretchability, high sensitivity, and adhesion, supporting wound healing and real-time strain monitoring. Furthermore, Yu et al. [149] developed a molybdenum disulfide-gelatin methacryloyl (MoS_2_-GelMA) hydrogel-based TENG skin patch. Leveraging MoS_2_’s conductivity and photothermal properties, the device accelerated tissue regeneration through combined electrical stimulation and photothermal therapy.

Despite their advantages—flexibility, biocompatibility, and ionic conductivity—hydrogels suffer from limited durability, dehydration, and mechanical mismatch with packaging materials, which can hinder long-term TENG performance. Overcoming these limitations is essential for advancing hydrogel-based TENGs.

##### Aerogels

Aerogels are ultra-light, porous materials synthesized by substituting the liquid phase of a gel with gas, typically through supercritical drying, to preserve the gel’s nanostructure. Their low density and high surface area make them ideal for enhancing triboelectric output [149,150]. Aerogels have emerged as promising candidates in the design of TENGs for flexible sensing applications [150]. Their defining characteristics—extremely low density, high surface area, and nano-porous architecture—make them particularly suitable for applications requiring high sensitivity, mechanical resilience, and structural adaptability [151,152].

In the context of TENGs, aerogels can be tailored to exhibit both ionic and electronic conductivity by embedding nanomaterials like CNTs, graphene, and metal oxides [153,154,155]. These dopants form percolated networks within the aerogel matrix, enabling efficient charge transport and enhancing triboelectric performance [156]. The conductivity of aerogels is highly tunable, depending on the type and concentration of fillers, as well as the processing conditions used during synthesis [155].

Morphologically, aerogels possess a sponge-like, open-cell structure that allows for large mechanical deformation without structural collapse [155]. This feature is critical for maintaining consistent triboelectric output under cyclic loading, especially in wearable and robotic systems. The high porosity of aerogels also facilitates efficient contact electrification by increasing the effective surface area available for charge generation. Aerogel-based TENGs have been successfully used in electronic skin, pressure mapping, and soft robotic interfaces, where sensitivity and responsiveness are critical. Tan et al. [126] used silk fibroin (SF) aerogel as a friction layer in TENGs, achieving an open-circuit voltage of 365 V, a 6.5-fold increase over SF film-based devices. The porous structure significantly improved the contact area and output performance. Wang et al. [147] demonstrated the use of aerogels in wearable systems for medical and hygiene applications, highlighting their potential in self-powered health monitoring.

While aerogels offer lightweight, high-performance, and customizable properties, they also face challenges such as low mechanical strength, limited conductivity, and complex fabrication. Future research should focus on improving these aspects to expand aerogel applications in TENGs.

##### Ionic Gels

Ionic gels are formed by incorporating ionic liquids into a polymer network. Unlike hydrogels, which rely on water as the solvent, ionic gels utilize salts composed of cations and anions. These ionic liquids offer distinct advantages, including low volatility, high electrical conductivity, and a wide electrochemical window [157]. These properties make ionic gels highly suitable for electrochemical and energy harvesting applications. In TENGs, ionic gels provide adjustable ionic conductivity, mechanical flexibility, and thermal stability [158,159]. Zhu et al. [160] enhanced the ionic conductivity of an ionogel to 0.53 S/m by optimizing the content of amino-terminal hyperbranched polyamide (NH_2_-HBP). The resulting SI-TENG, coated with silicone rubber, operated stably across a wide temperature range (−80 °C to 250 °C) and achieved an open-circuit voltage of 247 V and a short-circuit current of 11.7 μA for a 3 cm × 3 cm device.

To address the flammability issue in transparent triboelectric materials, Kim et al. [161] developed flame-retardant ion-gel films that maintained voltage output while offering optical transparency and mechanical flexibility, making them ideal for wearable energy solutions. Similarly, Zhong et al. [162] introduced a core-sheath-structured implantable TENG, integrating ionogel electrodes with aramid fiber and 3D-printed silicone serving as the positive and negative friction layers, respectively. The device maintained triboelectric output at 200 °C, and with the help of a support vector machine (SVM) algorithm, it achieved 85.00% accuracy in weight classification and 90.38% accuracy in shape recognition, demonstrating potential for high-temperature object recognition and classification. Additionally, Wu et al. [163] developed a multifunctional triboelectric-piezoelectronic skin (TPES), integrating a capacitive pressure sensor (PPS) and TENG. Additionally, Wu et al. [164] presented a TPES, incorporating both a capacitive pressure sensor (PPS) and a TENG, capable of detecting pressures from 616.42 kPa to 0.6 MPa with a swift response time of 5.6 ms. Combined with a 1D convolutional neural network, TPES enabled gesture detection, material perception, and real-time interaction with virtual characters, opening new avenues for VR and intelligent human–machine interfaces. Table 3 summarizes the representative material compositions along with their corresponding output voltage and current values for each dielectric category.

To provide a comprehensive overview of the diverse gel materials employed in TENGs, the following table summarizes their classification based on network structure, matrix composition, and contained liquid phase (Table 4). Each gel type is evaluated in terms of its functional roles within TENG architecture, key advantages, limitations, and relevant sensing applications. This comparative analysis serves as a practical reference for material selection and design optimization in gel-based TENG systems, supporting the development of high-performance, flexible, and multifunctional sensing platforms.

### 3.2. Gel Network Formation and Crosslinking Mechanisms in Polymer Gels for TENGs

The structural integrity and functional adaptability of gel-based TENGs are fundamentally governed by the network architecture and crosslinking mechanisms of the polymer gels employed. These mechanisms are broadly categorized into physical crosslinking, such as hydrogen bonding, ionic interactions, and hydrophobic associations, and chemical crosslinking, including covalent bonding via imine formation, Schiff base reactions, and borate ester linkages. The choice and design of crosslinking strategies directly influence the gel’s elasticity, toughness, and triboelectric performance under mechanical deformation. Recent innovations have focused on dual-network hydrogels, which combine soft and rigid polymer chains to balance flexibility and mechanical strength. For example, Sun et al. developed a double-network hydrogel composed of polyacrylamide and alginate, achieving over 1500% stretchability, high transparency (>95%), and excellent biocompatibility. This hydrogel was used to fabricate a stretchable TENG (SH-TENG) capable of powering up to 50 LEDs and enabling self-powered raindrop visual sensing for smart vehicle systems [179]. Similarly, Zhang et al. reviewed various stretchable hydrogel-based TENGs for on-skin electronics, emphasizing their self-healing, high ionic conductivity, and continuous deformation tolerance, which are critical for wearable health monitoring and human–machine interfaces [180].

In addition to mechanical reinforcement, photoreactive crosslinkers have introduced a new dimension of spatiotemporal control in gel networks. These types of materials allow for dynamic tuning of gel stiffness and shape, facilitating adaptive sensing platforms and reconfigurable electronics. Another promising class of materials is hypercrosslinked polymers (HCPs), synthesized via Friedel–Crafts alkylation or polycondensation reactions. These gels exhibit extremely high surface areas, hierarchical porosity, and chemical stability, making them ideal for energy harvesting and gas sensing applications. Collectively, these advanced gel architectures—ranging from dual-network hydrogels to photoreactive and hypercrosslinked systems—offer enhanced mechanical resilience, environmental stability, and functional tunability. Their integration into TENGs not only improves energy conversion efficiency but also supports the development of self-powered, flexible, and sustainable sensing technologies for applications in wearable electronics, environmental monitoring, and biomedical diagnostics.

### 3.3. Materials and Additives Used to Enhance Conductivity in Polymer Gel-Based TENGs

Enhancing conductivity in polymer gel-based TENGs is essential for improving charge transfer efficiency, output performance, and sensing sensitivity. Conductivity in these systems can be achieved through ionic or electronic pathways, and is typically engineered by incorporating a variety of functional additives into the gel matrix. These additives include metal ions, conductive polymers, and nanomaterials, each contributing distinct advantages to the gel’s electrical and mechanical properties.

#### 3.3.1. Ionic Conductivity Enhancers

Ionic conductivity is a critical parameter in gel-based TENGs, especially when gels are used as electrodes or sensing layers. Enhancing ionic conductivity improves charge transport, signal sensitivity, and overall device performance [4]. Several strategies have been developed to optimize the ionic conductivity of gel matrices, primarily through ion doping [4,181], ionic liquid incorporation [182], and network engineering [183,184].

One of the most common approaches involves doping hydrogels with metal salts such as LiCl, NaCl, FeCl_3_, or CaCl_2_, which introduce mobile ions into the gel network and facilitate ion migration under mechanical stimulation [185,186,187]. For instance, Chen et al. developed a chitosan/acrylamide/LiCl hydrogel that achieved an ionic conductivity of 56.7 mS/cm, while also exhibiting excellent anti-freezing properties and mechanical flexibility. This hydrogel was integrated into a TENG for wearable speech recognition, achieving a sensitivity of 1.56 V/kPa and over 96% recognition accuracy through machine learning-assisted analysis [188].

Another effective method is the use of ionic liquids (ILs), which offer high ionic mobility, wide electrochemical windows, and thermal stability [189]. ILs such as EMIM][DCA] (1-ethyl-3-methylimidazolium dicyamide), [BMIM][BF_4_] (1-butyl-3-methylimidazolium tetraffluoroborate), and LiTFSI have been incorporated into organogels and ionogels to enhance conductivity and environmental tolerance [190,191]. These gels maintain performance across a broad temperature range and resist dehydration, making them suitable for long-term wearable and implantable applications [191,192,193,194,195].

Additionally, polymer network design plays a vital role. Introducing interpenetrating polymer networks (IPNs) or dynamic crosslinking can improve ion transport pathways and mechanical integrity [196,197]. For example, hydrogels based on PVA/PAM or PAA/GA matrices doped with ionic species have demonstrated rapid self-healing, high stretchability, and stable conductivity under strain [198,199].

In summary, the enhancement of ionic conductivity in gel matrices is achieved through a combination of chemical doping, ionic liquid integration, and network architecture optimization. These strategies not only improve the electrical performance of TENGs but also expand their applicability in extreme environments, real-time biosensing, and intelligent human–machine interfaces.

#### 3.3.2. Electronic Conductivity Enhancers

Electronic conductivity in gel matrices is essential for enabling efficient electron transport, particularly when gels are used as electrodes in TENGs [200]. Unlike ionic gels, which rely on ion migration, electronically conductive gels form percolation networks that allow for direct electron flow under mechanical stimulation. This is typically achieved by incorporating conductive polymers or nanomaterials into the gel matrix [201,202].

Common conductive polymers used include poly(3,4-ethylenedioxythiophene):polystyrene sulfonate (PEDOT:PSS), polyaniline (PANI), polypyrrole (PPy), and polythiophene [203,204]. These materials are known for their intrinsic conductivity, flexibility, and compatibility with soft substrates. In addition to polymers, carbon-based nanomaterials such as CNTs, graphene, and MXene nanosheets are frequently used to enhance electronic conductivity. These nanomaterials form interconnected networks within the gel, significantly improving charge transport and mechanical strength. For instance, MXene/PVA hydrogels have demonstrated enhanced conductivity and triboelectric output due to the formation of hydrogen-bonded crosslinks and microchannel-like structures that facilitate electron flow [205,206,207].

Moreover, hybrid strategies combining conductive polymers and nanomaterials are gaining traction. These approaches leverage the synergistic effects of both components to achieve superior conductivity, mechanical resilience, and environmental stability. Such hybrid gels are particularly useful in applications requiring long-term durability, high sensitivity, and multifunctionality, including electronic skin, bioelectronic interfaces, and soft actuators [208,209,210,211,212].

In summary, enhancing electronic conductivity in gel matrices involves the strategic incorporation of conductive polymers, nanomaterials, and hybrid structures. These materials not only improve the electrical performance of TENGs but also expand their applicability in wearable electronics, human–machine interfaces, and next-generation soft devices [213,214].

#### 3.3.3. Nanomaterial-Based Additives

Nanomaterials have emerged as powerful additives for enhancing both the electrical conductivity and mechanical robustness of gel matrices used in TENGs. Their high surface area, tunable morphology, and intrinsic conductivity make them ideal for forming percolation networks within polymer gels, thereby facilitating efficient charge transport and improving structural integrity under mechanical stress [58,215,216,217,218,219].

Commonly used nanomaterials include CNTs, graphene, MXenes, and metal nanoparticles such as silver (Ag) and gold (Au). These materials not only improve conductivity but also contribute to antibacterial properties, biocompatibility, and environmental stability, making them suitable for wearable and biomedical applications [218,219,220,221,222,223].

For example, silver nanowires (AgNWs) embedded in methacrylic alginate hydrogels achieved conductivities up to 1.54 × 10^5^ S/m, enabling high electrical performance under strain. The Ca^2+^ ions in the alginate matrix further supported cell proliferation and vascularization, demonstrating the hydrogel’s potential for bioelectronic and regenerative applications [220]. Similarly, lignosulfonate/PVA hydrogels doped with In situ reduced Ag nanoparticles reached conductivities of 3300 S/m. These hydrogels exhibited fast response times (<20 ms) and high sensitivity when used as strain sensors to monitor subtle human body movements such as pulses and vibrations [224].

In addition to metal-based nanomaterials, carbon-based additives like CNTs and graphene are widely used to reinforce gel networks. These materials enhance mechanical strength, stretchability, and fatigue resistance, while maintaining excellent conductivity. MXene nanosheets, with their layered structure and surface functional groups, offer additional benefits such as electromagnetic shielding, thermal stability, and self-healing capabilities when integrated into gel matrices [225,226,227,228].

Overall, the incorporation of nanomaterials into gel-based TENGs represents a versatile and effective strategy to achieve multifunctional performance, enabling applications in flexible electronics, biosensing, soft robotics, and intelligent healthcare systems.

#### 3.3.4. Hybrid and Composite Strategies

To overcome the limitations of single-component conductive gels, recent research has focused on hybrid and composite strategies that combine multiple types of conductive fillers and additives. These approaches aim to synergistically enhance both electrical conductivity and mechanical resilience, which are critical for the performance and durability of TENGs [229].

One prominent strategy involves the integration of MXene nanosheets with ionic salts and conductive polymers to form dual-network hydrogels [230]. These composite gels exhibit superior triboelectric output and mechanical toughness due to the formation of interconnected conductive pathways and dynamic crosslinking. For example, MXene/PVA hydrogels doped with borate salts have demonstrated enhanced conductivity and stretchability, making them suitable for wearable sensors and soft robotics applications [231,232]. Priya et al. emphasized the role of dual-network and nanocomposite hydrogels in addressing challenges related to robustness and conductivity, particularly in energy harvesting and bio-integrated sensing platforms [233]. These gels often combine a soft, stretchable matrix (e.g., PVA and PAM) with rigid or functional fillers (e.g., graphene, CNTs, and metal nanoparticles), resulting in materials that maintain high performance under cyclic loading and environmental stress. Moreover, hybrid gels can be engineered to exhibit self-healing, anti-freezing, and moisture-resistant properties by incorporating responsive components such as ionic liquids, tannic acid, or zwitterionic polymers. These multifunctional gels expand the operational range of TENGs, enabling their use in extreme environments, implantable devices, and smart textiles [234,235].

In summary, the integration of conductive materials and additives into polymer gels represents a cornerstone of modern TENG design. Hybrid and composite strategies not only improve electrical performance but also enhance mechanical durability and functional versatility, paving the way for advanced applications in wearable electronics, biomedical diagnostics, and sustainable energy systems

### 3.4. Impact of Conductivity on Triboelectric Performance and Sensing Sensitivity in Gel-Based TENGs

The ionic or electronic conductivity of polymer gels critically influences the triboelectric performance and sensing sensitivity of TENGs. Enhanced conductivity facilitates efficient charge transfer, reduces internal resistance, and improves the coupling between mechanical stimuli and electrical response, which is essential for high-performance energy harvesting and sensing applications.

#### 3.4.1. Influence on Triboelectric Output

Recent studies have demonstrated that increasing the conductivity of gel materials significantly boosts the triboelectric output. For instance, Huang et al. introduced a conductive hydrogel with exceptional stretchability by integrating PEDOT:PSS into a gelatin-acrylamide matrix.

The resulting TENG achieved an output voltage of 192 V, with an ultra-fast response time of 0.15 s, and a sensing range of 0–1690%, making it suitable for human motion monitoring [236]. The conductive network formed by PEDOT:PSS facilitated rapid electron transport, enhancing the triboelectric charge collection and reducing energy loss. Similarly, Ba et al. introduced a CaCl_2_-doped cellulose nanofibril hydrogel that leveraged both ionic and electronic conduction mechanisms. This dual-conductive system enabled superstable electrical output under varying humidity conditions, demonstrating the robustness of electron–ion coupling in maintaining consistent triboelectric performance [237].

#### 3.4.2. Enhancement of Sensing Sensitivity

Conductivity also directly affects the sensitivity and resolution of TENG-based sensors. Conductive gels with optimized ionic pathways can respond to minute mechanical deformations, enabling precise detection of pressure, strain, and vibration. For example, Lu et al. reviewed gel-based TENGs for flexible sensing and highlighted that ionic conductivity in hydrogels enhances the responsiveness to low-pressure stimuli, which is critical for applications in electronic skin, tactile sensors, and health monitoring [34]. Moreover, Zhao et al. demonstrated that doping PDMS with CNTs increased the electrical conductivity and tripled the output current compared to pure PDMS. This improvement translated into higher sensitivity in detecting mechanical inputs, making CNT-doped gels ideal for wearable biosensors and gesture recognition systems [34].

## 4. Morphological Topography and Correlation with Triboelectric Output

### 4.1. Surface Topography

The morphological design of gel-based TENGs is a critical determinant of their energy harvesting efficiency and mechanical adaptability. Surface topography—including roughness, patterning, and porosity—directly influences the triboelectric interface, while advanced fabrication techniques (lithography, templating, and 3D printing) enable precise control over these features. Understanding the correlation between surface morphology and triboelectric output is essential for optimizing device performance across diverse applications.

#### 4.1.1. Surface Roughness

**Figure 2:**
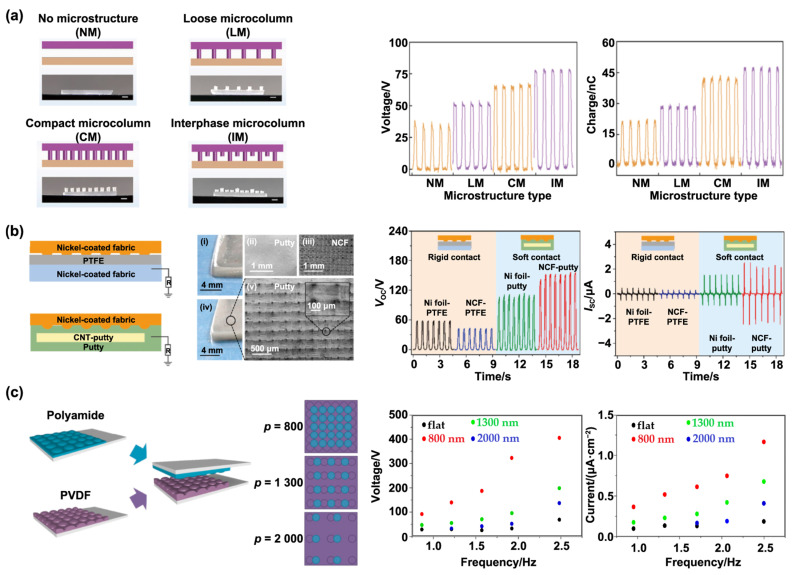
Effect of surface morphology on the output performance of TENGs. (**a**) Output comparison of TENGs with different microstructural designs: flat surface, loosely packed microcolumns, densely packed microcolumns, and interphase microcolumns. Adapted with permission from [240]. Copyright © 2021 American Chemical Society. (**b**) Soft solid−solid contact and shape adaptability of the SS-TENG; Digital photos of the SS-TENG after contact with nickel foil (**i**) and nickel-coated fabric (NCF) (**iv**); SEM images of the surface of putty contacted with nickel foil (**ii**) and NCF (**v**); (**iii**) SEM image of the surface of the NCF; Voltage and current outputs of TENGs using soft putty and rigid PTFE materials in contact with planar nickel foil and microrough NCF. Adapted with permission from [241]. Copyright © 2019 American Chemical Society. (**c**) Electrical performance of polyamide and PVDF-based TENGs with and without semi-ellipsoidal surface arrays. Adapted with permission from [242]. Copyright © 2021 Elsevier Ltd.

Enhancing the surface roughness of triboelectric materials is a fundamental strategy for improving the output performance of TENGs, as rough surfaces increase the specific surface area and effective contact area between triboelectric layers, thereby boosting frictional interaction, charge transfer, and surface charge density. This leads to a stronger electrostatic potential difference between electrodes; however, excessive roughness can elevate the electric field strength to the point of air breakdown, limiting further performance gains. To address this, researchers have explored five key strategies: selecting triboelectric materials with high polarity, modifying surfaces chemically or physically, creating material composites, designing micro-/nano-scale structures, and engineering electrodes for optimal charge collection [238]. Among these, micro-/nano-structuring has shown exceptional promise, with geometric arrays, bionic textures, and wrinkled morphologies (as shown in Figure 2a–c) significantly outperforming flat surfaces due to enhanced roughness and charge-trapping capabilities [239].

The data in Figure 2c clearly demonstrate that surface roughness plays a critical role in enhancing triboelectric performance. The variable “*p*” in Figure 2c refers to the pitch of the surface microstructures—specifically, the distance between adjacent features in nanometers (e.g., ridges, pyramids, or other patterns) on the triboelectric surface.

The pitch (p) is a critical design parameter in surface micropatterning because it influences the effective contact area, charge density, and mechanical deformation behavior of the triboelectric layers. The 800 nm nanostructured surface consistently yielded the highest current output across all frequencies—indicating an optimal balance between surface area enhancement and charge trapping efficiency. In contrast, the flat surface shows the lowest output, reaffirming the importance of surface engineering, while the 1300 nm and 2000 nm structures show moderate improvements, possibly limited by reduced mechanical contact or air breakdown effects. These structures can be fabricated using techniques such as template molding, electrospinning, etching, and printing, allowing for precise control and integration into wearable, flexible, and self-powered systems where both high performance and mechanical adaptability are essential.

These structures can be fabricated using diverse techniques, including template molding, electrospinning, etching, and printing, allowing for tailored designs based on material properties and application needs. This morphological modification not only boosts triboelectric efficiency but also enables scalable and versatile integration into wearable and flexible energy harvesting systems.

#### 4.1.2. Micro/Nano-Patterning

Micro- and nano-patterning introduces controlled topographical features—such as grooves, ridges, domes, and hierarchical wrinkles—that concentrate mechanical stress and promote localized deformation. These features enhance triboelectric charge generation by increasing dynamic contact area and improving mechanical responsiveness. Patterned gels are particularly advantageous in wearable electronics, where conformability to skin and sensitivity to motion are essential [243].

A novel design approach to improve the efficiency of TENGs involves enhancing both the triboelectric effect and capacitance change through micro-patterned thin films. The TENG was fabricated using PDMS films patterned with microstructures—lines, cubes, and pyramids—produced through photolithography with silicon wafer molds [244]. These structures were integrated into a sandwich device composed of indium tin oxide (ITO)-coated polyethylene terephthalate (PET) substrates, resulting in a significant performance boost.

**Figure 3:**
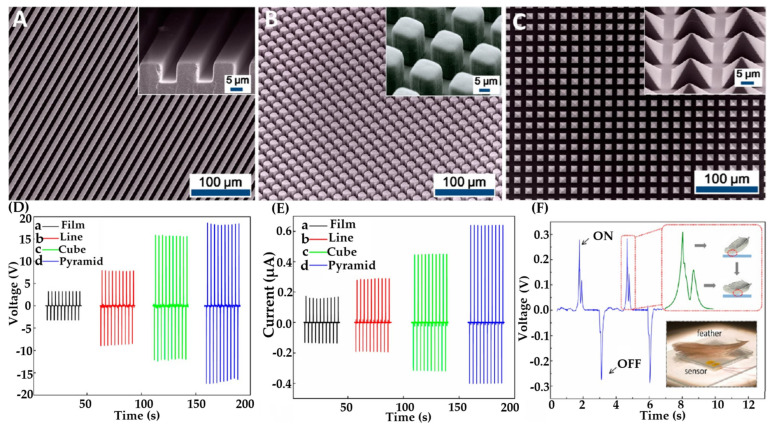
Surface patterning and pressure sensing performance of TENGs. (**A**–**C**) Scanning electron microscope (SEM) images of PDMS friction layers fabricated via traditional photolithography, featuring (**A**) line, (**B**) cube, and (**C**) pyramid microstructures. (**D**,**E**) Output voltage and current comparison between flat and patterned surface configurations. (**F**) Pressure sensing performance of the device triggered by a feather. The top inset illustrates the generation of a double-peak signal upon feather placement, while the bottom inset shows a photograph of the feather-induced sensor. Adapted with permission from [244]. Copyright © 2012 American Chemical Society.

As shown in Figure 3A–C, SEM images illustrate the distinct surface morphologies of the PDMS friction layers. Figure 3D–F present the corresponding electrical outputs, showing that the pyramid-patterned surface generated an open-circuit voltage of 18 V and a current of 0.7 μA—approximately four times greater than those of the flat PDMS films. Building on this, Sun et al. utilized natural leaf molds to imprint rich micro-nano textures onto PDMS surfaces, demonstrating that biomimetic patterning can further enhance triboelectric performance [245]. Additionally, Rasel et al. developed a cost-effective method using sandpaper templates to create complementary microstructures on PDMS without surfactants or vacuum systems. Their study showed that sandpaper-induced roughness significantly increased surface area and dielectric properties, leading to improved output stability and charge density [246]. These findings collectively underscore the critical role of surface morphology in optimizing TENG performance through scalable and versatile fabrication techniques.

#### 4.1.3. Porosity

**Figure 4:**
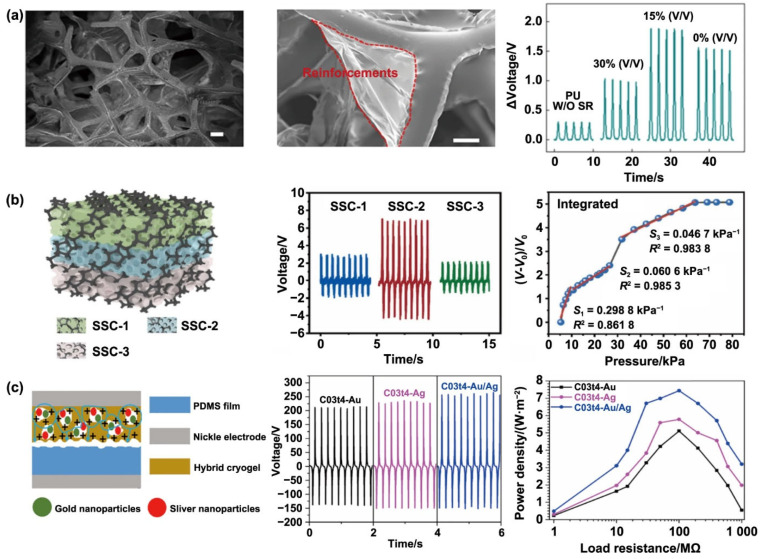
Influence of porous structures on the performance of TENGs. (**a**) Carbon nanotube-coated polyurethane (PU) sponges and silicone composites (SSCs) (Scale bars, 100 μm). Adapted with permission from [250]. Licensed under CC BY 4.0. (**b**) Structural design and output characteristics of a single-electrode TENG featuring a hierarchical porous architecture. Adapted with permission from [251]. Copyright © 2024 Elsevier Ltd. (**c**) Porous TENG incorporating silver and gold nanoparticles for enhanced performance. Adapted with permission from [252]. Copyright © 2020 John Wiley & Sons Ltd.

Porous gels provide enhanced breathability, compressibility, and responsiveness, which make them ideal candidates for wearable triboelectric devices. Porous architectures in gel matrices offer dual advantages: increased surface area for contact electrification and enhanced mechanical compliance [243]. The presence of interconnected pore networks allows for rapid deformation and recovery, which is essential for dynamic sensing applications such as gait monitoring, pulse detection, and joint movement analysis [247,248]. Internal and surface porous structures significantly enhance the performance of TENGs by increasing contact area, charge density, and mechanical adaptability. Based on pore size, porous materials are classified into micropores (<2 nm), mesopores (2–50 nm), and macropores (>50 nm), each influencing triboelectric behavior in distinct ways [249]. A porous patch TENG, fabricated by immersing PU sponges in diluted silicone rubber (Ecoflex-00-30), exhibited tunable pore sizes and mechanical properties, with a sensitivity of 0.21 kPa^−1^ and a detection range reaching 50 kPa (Figure 4a) [250].

Similarly, a single-electrode TENG, constructed from carbon nanotube-coated PU sponges and silicone composites (SSCs), is shown in Figure 4b. The term SSC 1–3 refers to different configurations of carbon nanotube-coated polyurethane (PU) sponges and silicone composites (SSCs) used as the active layers in the sensor architecture. Each SSC variant (SSC-1, SSC-2, and SSC-3) represents a different tuning of this porous architecture, allowing the sensor to achieve ultra-high sensitivity (up to 1622 kPa^−1^) and a wide detection range (up to 160 kPa) with excellent conductivity and a rapid response time [251]. Furthermore, Figure 4c presents a systematic enhancement of TENG performance through the incorporation of metal nanoparticles—silver (Ag), gold (Au), and their bimetallic combination (Ag/Au)—into a lactic acid and acrylamide-based polymer P(LA-AAm)] cryogel matrix [252]. Under identical testing conditions, silver nanoparticles (C03t4-Ag) yielded higher output (VOC = 233.85 V) than gold (C03t4-Au), attributed to silver’s greater tribopositivity and larger particle size, which increased surface roughness and effective contact area. The bimetallic hybrid cryogel (C03t4-Ag/Au) further improved performance (VOC = 262.14 V) due to synergistic charge generation and enhanced conductivity. Power output analysis under varying external load resistances revealed a peak power density of 7.44 W/m^2^ at 100 MΩ, which originates from the optimal balance between current and resistance, and can be described by the equation (P = I^2^R/A). This peak is physically driven by the enhanced surface morphology and charge transfer efficiency of the Ag/Au hybrid system, where silver contributes higher triboelectric activity and gold adds structural stability. The combination increases electron mobility and effective contact area, resulting in higher current output under optimal load conditions. These findings confirm the superior energy harvesting capability of the hybrid cryogel and its potential for further optimization in wearable and flexible TENG applications.

While the integration of Ag/Au nanoparticles into the P(LA-AAm) cryogel matrix significantly enhances triboelectric output and achieves a peak power density of 7.44 W/m^2^ under optimal load conditions, the work presents certain limitations. The performance evaluation is primarily conducted under controlled laboratory settings, with limited assessment of long-term mechanical durability, environmental stability (e.g., humidity and temperature fluctuations), and real-world applicability in wearable systems. Additionally, the use of noble metals like silver and gold, though effective, may pose cost and scalability challenges for large-scale or commercial deployment. Addressing these aspects in future studies would be essential to fully realize the practical potential of this high-performance hybrid cryogel-based TENG platform.

These examples underscore how porous architectures—whether engineered through freeze-drying, foaming, or self-assembly—enable multifunctional enhancements in TENGs, making them ideal for wearable sensors, energy harvesting, and biomedical applications.

#### 4.1.4. Layered Architectures and Synergistic Design

Beyond porosity, layered architectures—particularly sandwich-like configurations composed of alternating conductive and insulating layers—have been shown to optimize charge separation and mechanical resilience. These designs enable consistent electrical output under varying mechanical loads and are increasingly integrated into multifunctional platforms for energy harvesting and physiological monitoring. For instance, hybrid gel systems incorporating conductive hydrogels and elastomeric insulators have shown promise in applications ranging from self-powered biosensors to smart textiles [253].

**Figure 5:**
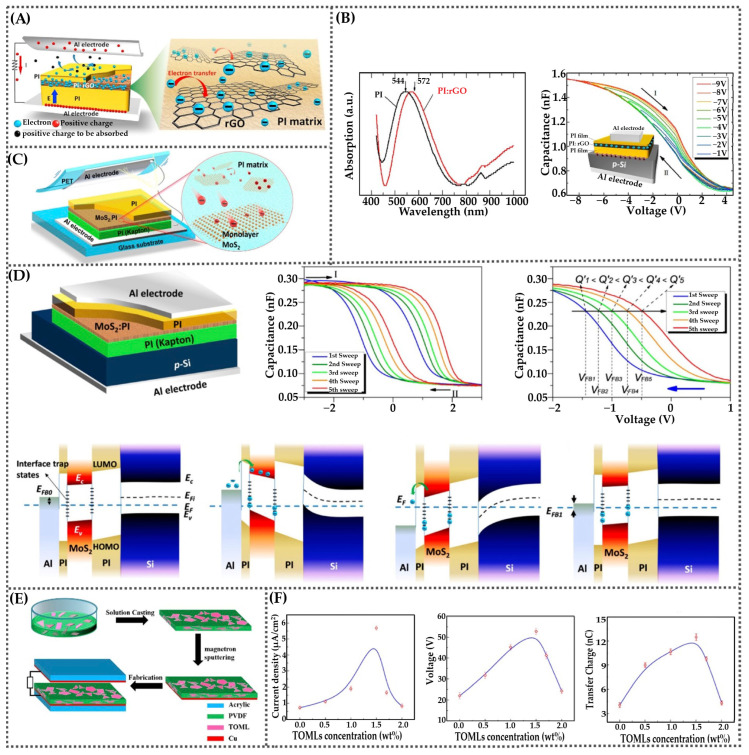
Nanocomposite-based TENGs and their electrical performance. (**A**) TENG device fabricated using a polyimide (PI) and reduced rGO composite. (**B**) Capacitance–voltage (C–V) characteristics of the Al/p-Si/PI/PI:rGO/PI/Al multilayer structure. Adapted with permission from [254]. Copyright © 2017 Elsevier Ltd. (**C**) TENG incorporating a monolayer MoS_2_ film as an electron-trapping layer. (**D**) Schematic representation of the PI: MoS_2_-based TENG configuration. Adapted with permission from [255]. Copyright © 2017 American Chemical Society. (**E**) TENG utilizing PVDF/TOML nanocomposite films. (**F**) Maximum electrical output observed at 1.5 wt% TOML concentration. Adapted with permission from [256]. Copyright © 2018 Elsevier Ltd.

The integration of composite materials—particularly 2D layered structures—into triboelectric layers has emerged as a powerful strategy to enhance TENG performance by introducing electron-trapping sites and modifying surface chemistry. These 2D materials, such as graphene, MoS_2_, and BaTiO_3_, possess high surface area and in-plane stability, making them ideal for charge trapping. Wu et al. (2017) demonstrated that incorporating reduced rGO into polyimide (PI) films significantly boosted output, with the PI:rGO composite achieving a power density of 6.3 MW/m^2^—30× higher than pristine PI—by preventing charge recombination and enhancing electron density (Figure 5A,B) [254].

Similarly, Wu et al. (2017) used monolayer MoS_2_ embedded in PI to create a floating-gate MIS device, where the electron-trapping effect of MoS_2_ led to a 120× increase in peak power density, as confirmed by C–V curve shifts under voltage loading (Figure 5C,D) [255]. Expanding on this, Wen et al. (2018) fabricated transparent TENGs using PVDF/TOML nanocomposites, where titania monolayers (TOMLs) enhanced dielectric properties and electron capture, resulting in a maximum output of 52.8 V and 5.7 µA at 1.5 wt% TOML—2.4× and 7.8× higher than pristine PVDF (Figure 5E,F) [256]. These studies collectively highlight the effectiveness of 2D and nanocomposite materials in boosting TENG output through enhanced charge trapping, dielectric modulation, and structural synergy.

The correlation between surface morphology and triboelectric output is well-established in gel-based TENGs. The strategic manipulation of roughness, patterning, and porosity enables the development of devices with enhanced sensitivity, durability, and multifunctionality. These insights are critical for advancing next-generation self-powered systems tailored for biomedical monitoring, environmental sensing, and interactive electronics. Future research is expected to focus on bioinspired designs and scalable fabrication techniques that integrate morphological and material innovations for optimized triboelectric interfaces.

## 5. Sensing Applications of Gel-Based Triboelectric Nanogenerators

### 5.1. Health Monitoring

Continuous health monitoring is a cornerstone of modern healthcare, enabling early disease detection, personalized treatment, and proactive health management. The convergence of flexible electronics, wireless communication, and self-powered sensing technologies has led to the development of TENGs integrated with gel materials, which offer real-time, non-invasive monitoring of physiological signals. These gel-based TENGs are capable of detecting vital biomarkers such as respiration rate, sweat composition, blood pressure, and tremor activity, thereby supporting a wide range of biomedical applications [257].

#### 5.1.1. TENG-Integrated Sweat Monitoring

**Figure 6:**
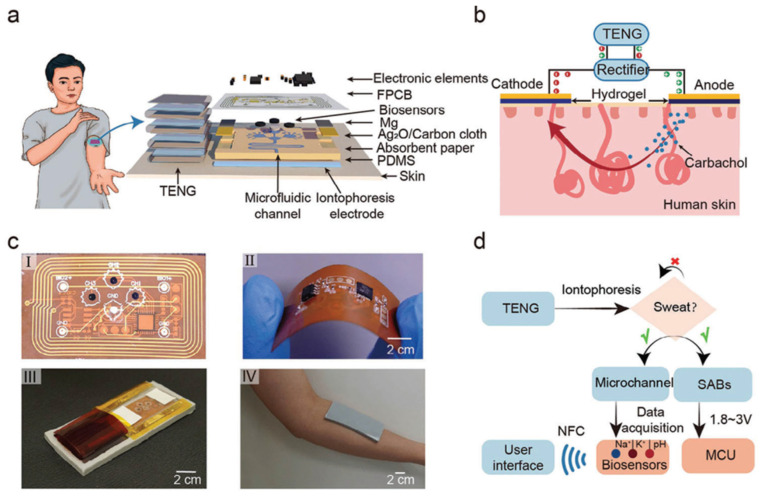
Overview and structural components of the SEMS system. (**a**) Conceptual illustration of the wearable SEMS designed for sweat stimulation in sedentary individuals. (**b**) Mechanism of sweat induction using TENG technology. (**c**) Photographs of SEMS device components: I. Microcircuit integrated with biosensors. II. Flexible printed circuit board (FPCB) demonstrating high bendability. III. PDMS encapsulation of the TENG and microcircuit. IV. Device securely adhered to the skin. (**d**) Functional workflow diagram outlining SEMS operation. Adapted with permission from [259]. Copyright © 2023 Wiley.

Sweat is a rich source of physiological information, containing electrolytes, metabolites, and trace elements that reflect the body’s health status [258]. Maintaining electrolyte balance is crucial for overall health, particularly in older adults who are more susceptible to conditions such as diabetes, kidney disease, and dehydration. Recent advances in wearable biosensors have enabled non-invasive monitoring of electrolytes through sweat analysis, offering a practical alternative to traditional diagnostic methods. Recently, Guoqiang Xu et al. have developed a wearable system that integrates TENGs to enhance sweat-based health monitoring (Figure 6a–d) [259]. TENGs are energy-harvesting devices that generate electricity from simple body movements, such as tapping or walking. This energy is used to stimulate sweat production and power biosensors, making the system particularly useful for individuals with low sweat output, such as the elderly or sedentary patients.

The system includes flexible sensors that detect key biomarkers—sodium (Na^+^), potassium (K^+^), and pH—with high sensitivity and stability. These sensors use advanced materials like PEDOT:PSS and polyaniline to ensure accurate and reliable readings. To improve sweat collection and sensor performance, hydrogels are added. These soft, water-rich materials help maintain skin contact and support ion movement, which enhances sensing efficiency. With TENG-induced electrical stimulation, sweat can be collected in just 8 min using carbachol, compared to 36 min without stimulation. The system also features a skin-attached supercapacitor-based battery (SAB) and a near-field communication (NFC) module, allowing real-time data collection and wireless transmission to mobile devices. The sensors perform consistently across different body locations and users, demonstrating the system’s robustness and adaptability.

#### 5.1.2. Glucose Monitoring for Diabetes Management

**Figure 7:**
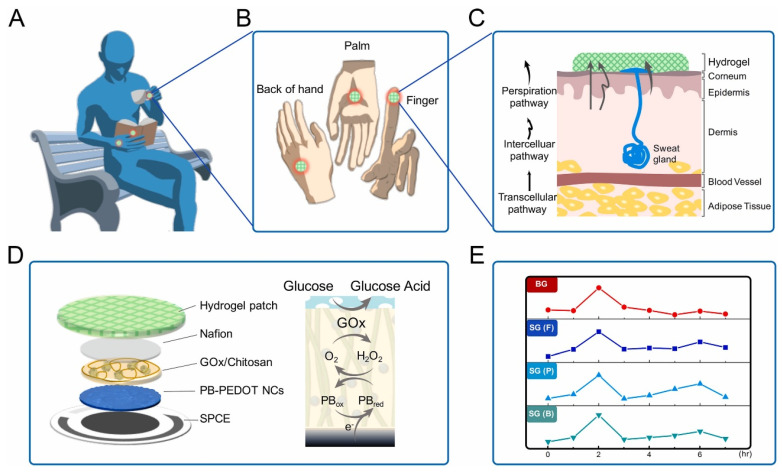
Design and operational mechanism of a natural sweat-sampling glucose sensor. (**A**) Schematic of a hydrogel patch designed for passive sweat collection during resting conditions. (**B**) Representative wearable positions—finger, palm, and back of the hand—for effective sweat sampling. (**C**) Preferential glucose transport pathways through hydrogel interfaces. (**D**) Layered structure of the enzymatic PB–PEDOT nanocomposite electrode and the glucose oxidase (GOx) reaction mechanism with the Prussian Blue (PB) probe. (**E**) Real-time monitoring of sweat glucose levels without external stimulation at various body locations: finger (F), palm (P), and back of hand (B). Reproduced with permission from [260]. Copyright © 2022 Elsevier Ltd.

Regular glucose monitoring is crucial for effective diabetes management and reducing the risk of associated complications. Lin et al. introduced a wearable hydrogel-based glucose sensor designed to monitor glucose levels in sweat without requiring invasive procedures or artificial sweat stimulation (Figure 7A–E) [260]. The device integrates a multilayer structure composed of a conductive hydrogel (PB–PEDOT), a glucose oxidase (GOx) sensing layer, and a Nafion protective membrane. This configuration enables the patch to absorb naturally secreted sweat directly from the skin, particularly from the hand, and transport it through a continuous hydrophilic pathway that connects the sweat gland to the sensor interface. As sweat diffuses into the hydrogel, glucose molecules are enzymatically converted by GOx, producing hydrogen peroxide (H_2_O_2_), which is then electrochemically reduced by the PB layer at low overpotential. This reaction generates a measurable signal corresponding to glucose concentration. The sensor demonstrated a broad linear detection range (6.25 μM to 0.8 mM, R^2^ = 0.992) and a low detection limit of 4 μM in phosphate-buffered saline, confirming its sensitivity and selectivity. The study highlights the potential of hydrogel-based platforms for comfortable, real-time glucose monitoring and their suitability for integration into wearable health technologies.

#### 5.1.3. Respiratory Monitoring and Air Filtration

**Figure 8:**
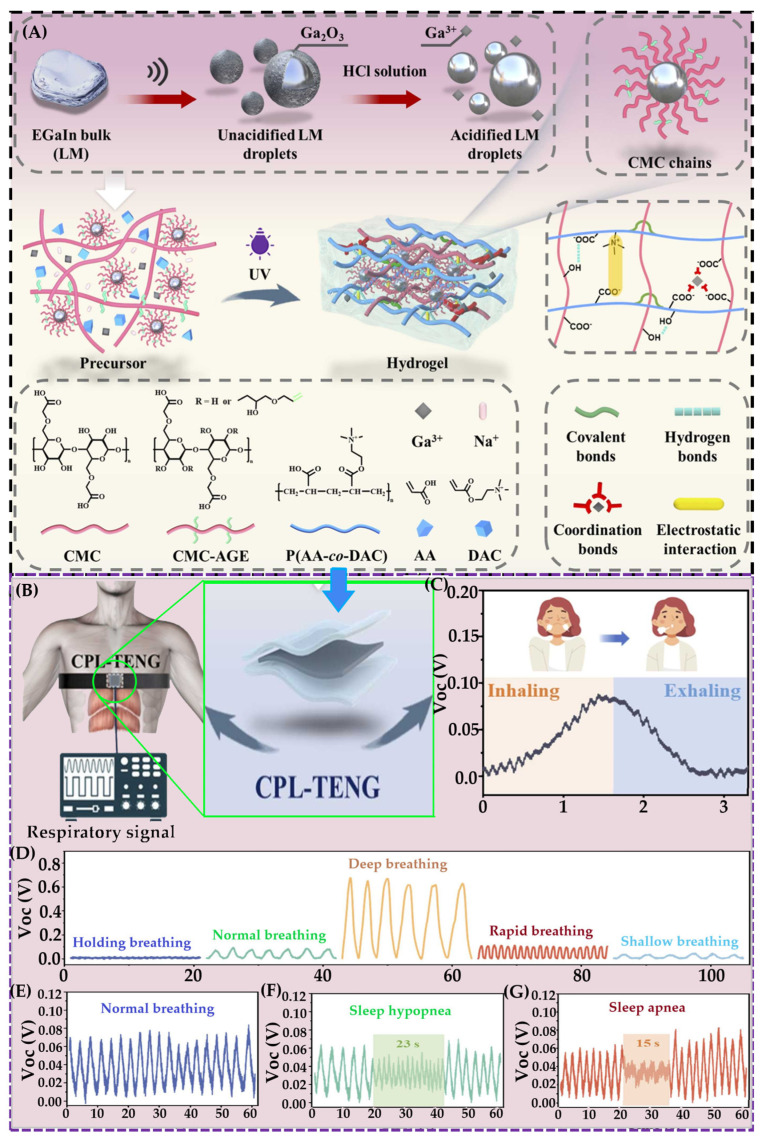
Schematic representation and performance of a hydrogel-based triboelectric sensor for respiratory monitoring. (**A**) Preparation and multifunctional applications of the CMC/P(AA-co-DAC)/NaCl-LM hydrogel. (**B**) Working principle of respiratory signal detection using the hydrogel-based sensor. (**C**) Illustration of a complete respiratory cycle, including inhalation and exhalation phases. (**D**) Real-time voltage signals corresponding to different respiratory conditions. (**E**–**G**) Electrical responses during normal breathing, sleep hypopnea, and sleep apnea, respectively. Adapted with permission from [261]. Copyright © 2025 Elsevier Ltd.

Respiration rate is a vital indicator of general health and respiratory conditions. Yang et al. introduce a self-powered respiratory monitoring system based on a conductive hydrogel-doped triboelectric nanogenerator (CPL-TENG), designed for real-time, non-invasive detection of respiratory signals (Figure 8A–G) [261]. The CPL-TENG utilizes a novel double-network hydrogel composed of carboxymethyl cellulose (CMC), poly(acrylic acid-co-acryloyloxyethyltrimethyl ammonium chloride) [P(AA-co-DAC)], NaCl, and oxide-free liquid metal (LM). This hydrogel matrix is engineered through synergistic interactions—hydrogen bonding, metal coordination, and electrostatic forces—resulting in excellent stretchability, conductivity, self-healing, and adhesion. LM droplets are stabilized by bio-macromolecules and deoxidized using HCl to enhance dispersion and electrical performance. Integrated into a wearable configuration, the CPL-TENG demonstrates high electrical output (peak voltage of 28 V) and mechanical stability over 1000 cycles. When applied to the chest, the device accurately captures respiratory signals by converting biomechanical deformation into voltage outputs, effectively distinguishing breathing patterns and detecting obstructive sleep apnea-hypopnea syndrome (OSAHS), with signal amplitude reductions of 39% during hypopnea and 85% during apnea.

#### 5.1.4. Air Filtration

**Figure 9:**
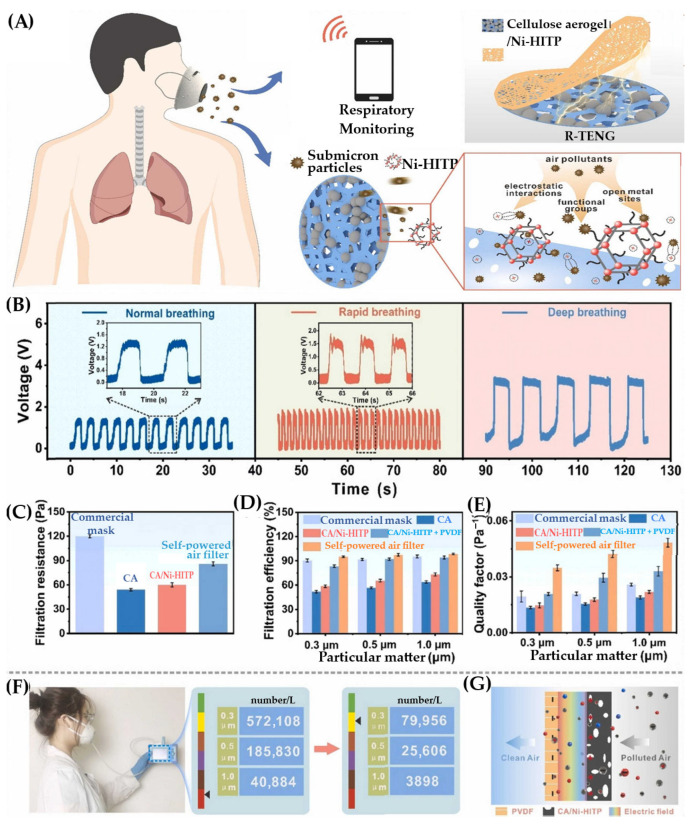
Design and performance of a self-powered air filtration system based on TENGs. (**A**) Schematic illustration of submicron particle capture using a CA/Ni-HITP composite. (**B**) Real-time voltage signals corresponding to various respiratory states. (**C**) Pressure drop comparison among commercial mask (CA), CA/Ni-HITP, and the self-powered air filter during filtration. (**D**,**E**) Filtration efficiency and quality factor (QF) of different configurations: commercial mask, CA, CA/Ni-HITP, CA/Ni-HITP+PVDF, and the self-powered air filter. (**F**) Quantification of submicron particles inside and outside the mask after 2 h of wear. (**G**) Schematic of the filtration mechanism enabled by the self-powered air filter. Adapted with permission from [263]. Copyright © 2022 Elsevier Ltd.

Recent advancements in wearable electronics have enabled multifunctional devices that combine health monitoring with environmental protection. Fu et al. developed a respiration-driven triboelectric nanogenerator (R-TENG) integrated into a facemask, offering simultaneous air filtration and respiratory signal tracking through a novel conductive cellulose aerogel/metal-organic framework (Ni-HITP) composite paired with a PVDF film (Figure 9A–G) [262]. The device utilizes a conductive cellulose aerogel/metal-organic framework (Ni-HITP) composite as the triboelectric and filtration material, paired with a polyvinylidene fluoride (PVDF) film. Fabricated via an In situ green synthesis method, the composite exhibits a porous network structure that enables efficient particle removal—achieving filtration efficiencies of 98.4% for PM1.0, 97.3% for PM0.5, and 95.0% for PM0.3—while maintaining a low pressure drop of 86 Pa. The R-TENG generates electrical signals in response to breathing-induced airflow, allowing real-time monitoring of respiratory patterns without external power. This dual-function system demonstrates the potential of cellulosic triboelectric materials in developing breathable, self-powered wearable healthcare devices for environmental protection and medical diagnostics.

#### 5.1.5. Tremor Detection for Neurological Assessment

**Figure 10:**
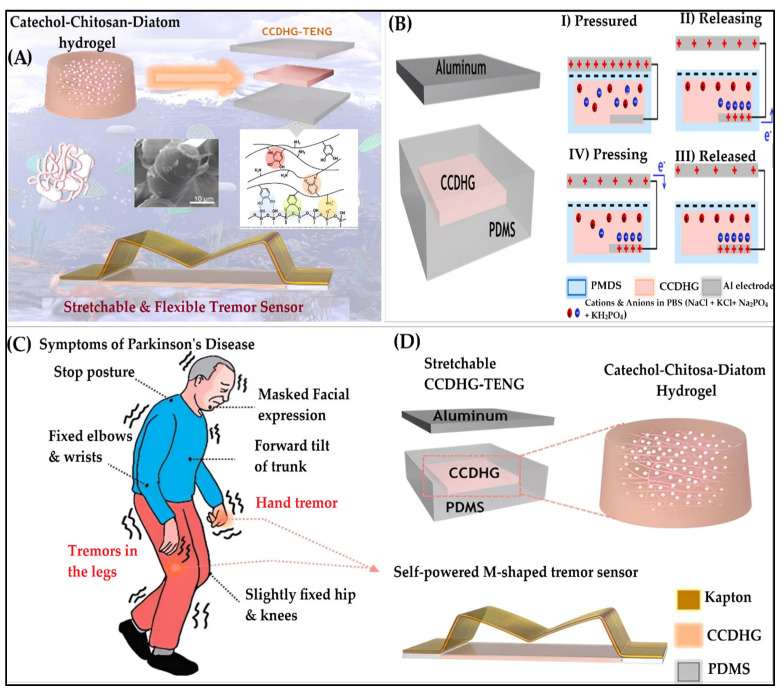
Design and mechanism of a biocompatible hydrogel-based triboelectric sensor for tremor detection. (**A**) Schematic illustration of a stretchable and biocompatible catechol–chitosan–diatom hydrogel (CCDHG) used in a self-powered tremor sensor. (**B**) Working principle of the CCDHG-TENG device. (**C**) Representative symptoms associated with Parkinson’s disease. (**D**) Schematic of the CCDHG-TENG integrated with a tremor sensing system. Adapted with permission from [128]. Copyright © 2021 Elsevier Ltd.

Neurological disorders such as Parkinson’s disease often manifest through tremors and low-frequency vibratory movements. To address this, a self-powered tremor sensor was developed by integrating a catechol–chitosan–diatom hydrogel (CCDHG) with an M-shaped Kapton film (Figure 10A–D) [128]. This study presents a multifunctional TENG based on a stretchable and self-healing hydrogel composed of catechol-modified chitosan and diatomaceous earth, designed for self-powered tremor sensing in Parkinson’s disease management. The hydrogel exhibits excellent mechanical properties, including high stretchability and rapid self-healing, attributed to dynamic catechol–metal coordination and hydrogen bonding. Integrated into a wearable TENG, the hydrogel enables efficient biomechanical energy harvesting and real-time motion sensing. The device demonstrates stable electrical output under repeated deformation and effectively captures tremor signals with high sensitivity and resolution. Its biocompatibility and adaptability to skin surfaces make it suitable for continuous, non-invasive monitoring of motor symptoms in neurodegenerative disorders. This work highlights the potential of bioinspired hydrogel-based TENGs in developing soft, self-powered diagnostic tools for personalized healthcare applications.

### 5.2. Environmental Monitoring

The integration of flexible sensor technologies into environmental monitoring systems has become increasingly vital in addressing global ecological challenges. Gel-based TENGs, owing to their inherent flexibility, responsiveness to external stimuli, and compatibility with diverse substrates, have emerged as promising candidates for self-powered environmental sensors. These systems are capable of detecting a wide range of environmental parameters, including humidity, temperature, gas concentration, and water quality, without the need for external power sources [27,35,263].

#### 5.2.1. Humidity Sensing via Aerogel-Based TENGs

**Figure 11:**
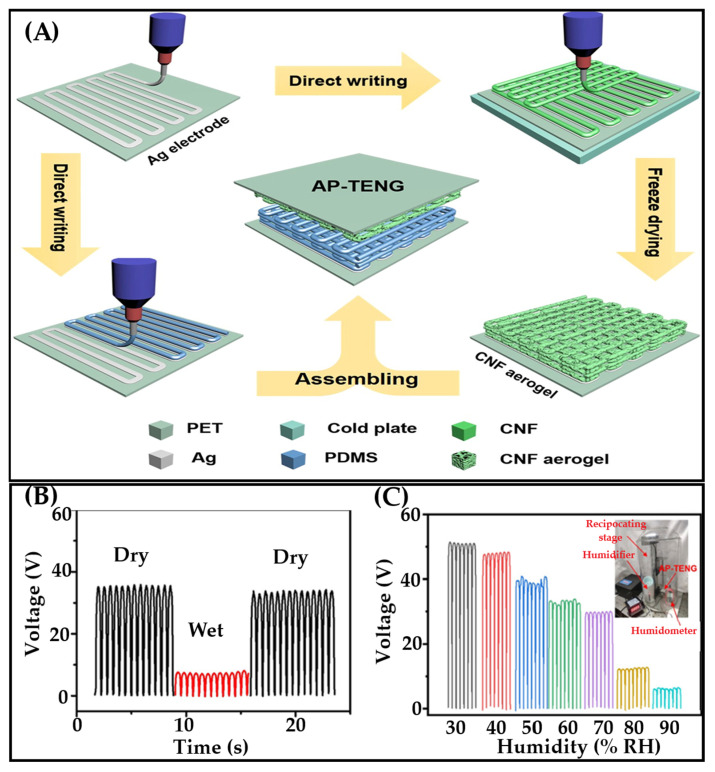
Gel-based TENGs for humidity sensing and environmental monitoring. (**A**) Fabrication process of the AP-TENG device. (**B**) Voltage signal response of the AP-TENG humidity sensor under dry and humid conditions. (**C**) Sensitivity of the AP-TENG humidity sensor across varying humidity levels. Panels (**A**–**C**) adapted with permission from [264]. Copyright © 2019 Elsevier Ltd.

Aerogels, characterized by their highly porous and lightweight structures, offer excellent water absorption capabilities, making them ideal for humidity sensing applications. A notable example is the all-printed 3D hierarchically structured cellulose aerogel-based TENG (AP-TENG), illustrated in Figure 11A–C. [264]. The micro/nanostructured cellulose aerogel enhances surface roughness and contact area, thereby improving triboelectric output. This device was successfully employed in a self-powered humidity sensor, demonstrating a response ratio of up to 5:1 by detecting moisture-induced surface potential variations. Such performance highlights the potential of aerogel-based TENGs in real-time atmospheric humidity monitoring.

#### 5.2.2. Fire Detection and Thermal Insulation

**Figure 12:**
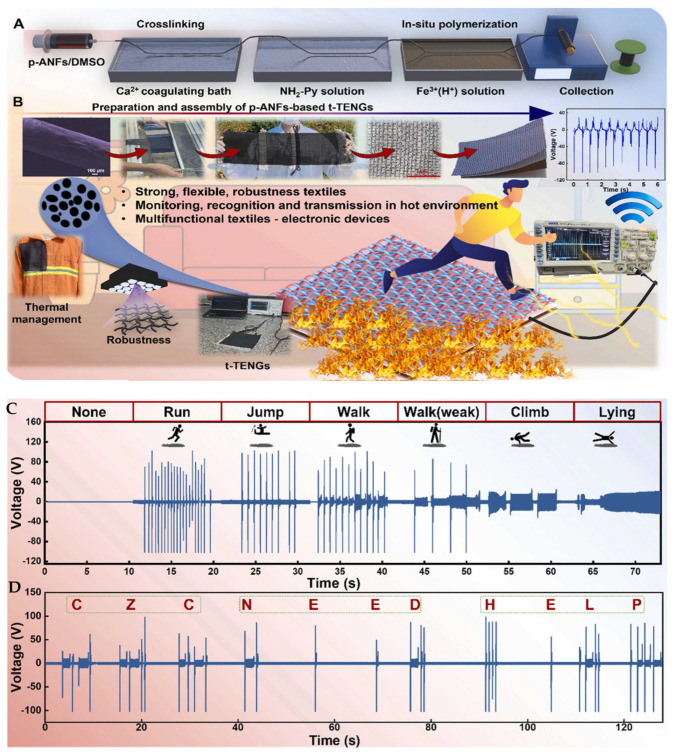
Fabrication and functional demonstration of p-ANF-based textile triboelectric nanogenerators (t-TENGs). (**A**) Preparation process of H_2_N-PPy/HMWCNTs/p-ANF porous films (PFs). (**B**) Structural design and working mechanism of lotus-root-like p-ANF-based t-TENGs. (**C**) Output voltage signals recorded from individuals in various motion states. (**D**) Conversion of mechanical energy into Morse code signals via frictional power generation from t-TENGs. Adapted with permission from [265]. Copyright © 2024 Elsevier Ltd.

Hydrogels exhibit superior thermal insulation and flame-retardant properties, which are critical for fire detection and safety applications. Chen et al. developed a high-performance textile-based triboelectric nanogenerator (t-TENG) using plasma-treated aramid nanofiber-based porous fibers (p-ANFs PFs), designed for multifunctional sensing and high-temperature escape monitoring (Figure 12A–D) [265]. The fabrication involved wet spinning and In situ polymerization of polyaminopyrrole (H_2_N-PPy) and hydroxylated multi-walled carbon nanotubes (HMWCNTs) within p-ANFs, resulting in a freeze-dried, hierarchically porous structure that enhanced thermal insulation and triboelectric output. The incorporation of nitrogen-rich H_2_N-PPy and amide bonds in p-ANFs significantly improved electron donation and sensor performance under extreme conditions. The t-TENG exhibited excellent flame retardancy, UV resistance, and thermal stability, maintaining 40% higher output at 120 °C compared to room temperature, and demonstrated resilience in sub-zero environments by increasing body temperature from 19 °C to 22 °C at −40 °C. Additionally, the device supported real-time escape signaling via Morse code and showed stable output during dynamic movements, making it a promising candidate for smart textiles in emergency response and harsh environmental applications.

#### 5.2.3. Marine Environmental Monitoring

**Figure 13:**
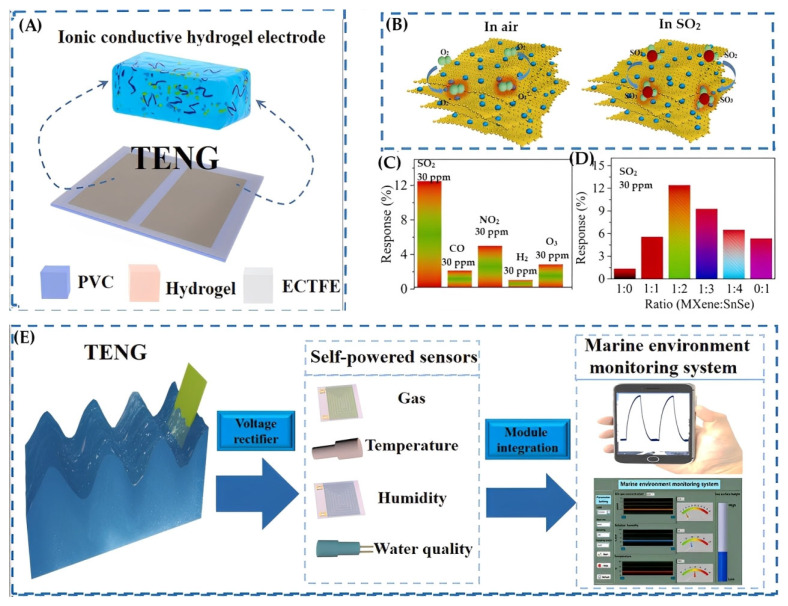
Structural design and sensing performance of a liquid–solid TENG for gas detection. (**A**) Photograph and schematic of the liquid–solid TENG device. (**B**) Schematic representation of the gas-sensing mechanism. (**C**) Selectivity profile of the sensor toward different gases. (**D**) Sensor response based on composite materials with varying mass ratios. (**E**) Schematic of a self-powered sensing system driven by the TENG. Adapted with permission from [2]. Copyright © 2022 Elsevier Ltd.

With increasing anthropogenic pressures on marine ecosystems, there is a growing need for robust and autonomous monitoring technologies. Gel-based TENGs have been adapted for marine applications through the development of liquid–solid interface TENGs, as depicted in Figure 13A–E [2]. This system comprises an ethylene chlorotrifluoroethylene (ECTFE) film, a PVA-ethylene glycol hydrogel electrode, and a PVC substrate. The device harnesses wave-induced variations in seawater contact area to generate electrical signals, enabling real-time monitoring of parameters such as SO_2_ concentration, temperature, humidity, and water quality. The system consists of an ethylene chlorotrifluoroethylene (ECTFE) film, a PVA–ethylene glycol hydrogel electrode, and a PVC substrate. It utilizes wave-induced changes in seawater contact to generate electrical signals, enabling real-time monitoring of parameters including SO_2_ concentration, temperature, humidity, and overall water quality. The self-powered nature of this system allows for continuous operation in remote aquatic environments, contributing to sustainable marine ecosystem management.

#### 5.2.4. Gas Detection

**Figure 14:**
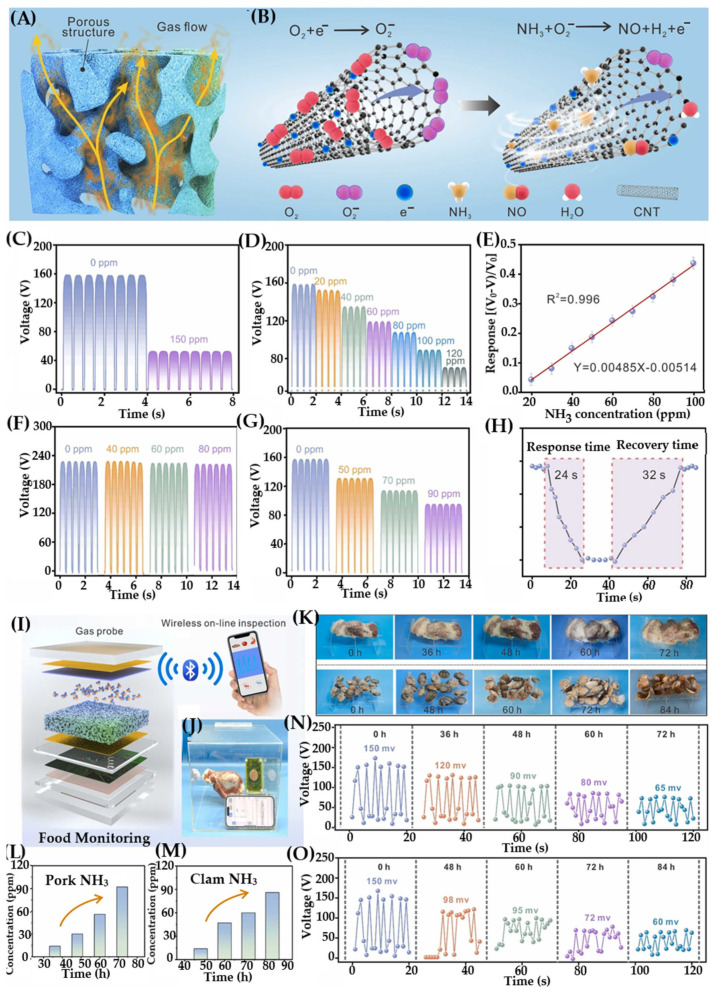
Ammonia sensing performance and practical application of the CPC-aerogel-based TENG. (**A**) Schematic representation of ammonia adsorption on the surface of the CPC-aerogel. (**B**) Illustration of the sensing mechanism based on triboelectric signal modulation induced by gas interaction. (**C**) Voltage output comparison at 0 ppm and 150 ppm ammonia concentrations. (**D**) Voltage response across a range of ammonia concentrations. (**E**) Linear relationship between the relative voltage change rate (|V_0_ − V|/V_0_) and ammonia concentration. (**F**) Comparative voltage output of FEP–nylon under varying ammonia levels. (**G**) Stability assessment of the sensor after 24 h of exposure. (**H**) Sensor response at 90 ppm ammonia concentration. (**I**) Conceptual diagram of the wireless sensing system for food quality monitoring using the triboelectric gas sensor. (**J**) Photograph of the CPC-aerogel-based sensor device. (**K**) Visual condition of food samples after different storage durations. (**L**,**M**) Ammonia concentration changes in pork and clam samples over time. (**N**,**O**) Corresponding wireless signal outputs for pork and clam, demonstrating real-time monitoring capability. Adapted with permission from [176]. Copyright © 2024 Elsevier.

Gel-based TENGs have also shown promise in gas sensing applications through the integration of porous and functionalized aerogel structures. In a recent study, Gao et al. developed a compressible triboelectric aerogel by engineering heterointerfaces between oxidized cellulose nanofibers (TCNF) and CNTs, forming a soft, gel-like matrix with hierarchical porosity (Figure 14A–O) [176]. The resulting aerogel exhibited a porosity of 97.23% and a 40-fold enhancement in compressive strength, enabling stable triboelectric output under repeated mechanical deformation. Functionalization with CNTs endowed the aerogel with selective sensitivity to ammonia gas, achieving a linear detection range from 20 to 150 ppm with a correlation coefficient of R^2^ = 0.996. The system was further integrated into a wireless monitoring platform for real-time detection of food spoilage, demonstrating its practical utility in environmental and safety applications. This work highlights the versatility of cellulose-derived gel-based TENGs in combining mechanical resilience, chemical selectivity, and self-powered operation for advanced gas sensing technologies.

### 5.3. TENG-Integrated Tactile Sensing

Gel-based TENGs have emerged as promising candidates for tactile sensing applications due to their inherent softness, stretchability, and biocompatibility. These properties make them ideal for integration into electronic skin (e-skin) platforms, enabling both biomechanical energy harvesting and pressure-sensitive sensing [173,266,267,268].

#### 5.3.1. Skin-like STIC for Tactile Communication

**Figure 15:**
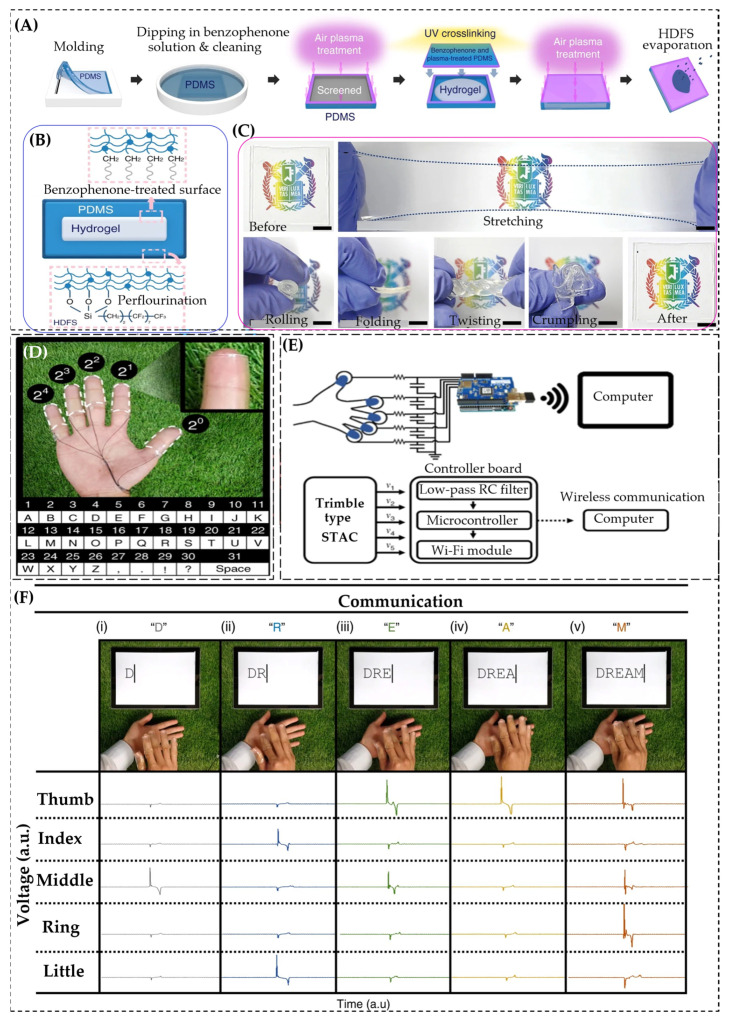
Fabrication and functional demonstration of a transparent and attachable ionic communicator (STAIC) based on a hydrogel–elastomer triboelectric interface. (**A**) Schematic illustration of the fabrication process, including chemical anchoring of the hydrogel to PDMS via benzophenone treatment and surface modification with HDFS for enhanced triboelectric output and self-cleaning functionality. (**B**) Cross-sectional view of the STAIC structure, showing the robust interface between the hydrogel and elastomer layers. (**C**) Mechanical deformation tests demonstrating the device’s flexibility under stretching, rolling, folding, twisting, and crumpling, followed by recovery. (**D**) Real-time wireless communication setup with STAICs mounted on fingers and connected to a microcontroller programmed with binary-coded inputs. (**E**) Block diagram of the STAIC-based communication system, comprising an RC low-pass filter, microcontroller, and Wi-Fi module. (**F**) Demonstration of gesture-based input using finger touches to transmit the word “DREAM,” validating the system’s capability for interactive communication. Adapted with permission from [36]. Copyright © 2018 Nature.

The development of transparent and skin-attachable tactile sensors has significantly advanced the field of wearable electronics. In a notable study, Lee et al. introduced a soft, transparent ionic communicator (STAIC) by integrating a hydrogel-based triboelectric nanogenerator with a PDMS elastomer, achieving high optical transmittance and mechanical flexibility suitable for on-skin applications (Figure 15) [36]. The hydrogel electrode was chemically anchored to the elastomer substrate using benzophenone treatment, ensuring robust interfacial bonding and long-term durability. To enhance surface properties, the PDMS layer was functionalized with HDFS, imparting self-cleaning capabilities and improving triboelectric output. The STAIC demonstrated excellent tactile sensitivity, enabling real-time gesture recognition and human–machine interaction through ionic signal transmission. This work highlights the potential of gel-based TENGs in developing multifunctional, transparent, and self-powered tactile sensors for next-generation wearable communication systems.

Recent studies have further expanded the potential of hydrogel-based TENGs by incorporating self-healing capabilities, which are crucial for long-term reliability in wearable applications. For instance, Li et al. [269] developed a poly(AMPS-co-AA-co-DMAPMA)/GO/Laponite hydrogel TENG that exhibited ultra-stretchability and full recovery of electrical output after mechanical damage. Similarly, Kaymazlar et al. [270] reported a borax-crosslinked PVA hydrogel TENG with dynamic hydrogen bonding and borate ester linkages, enabling rapid and repeatable self-healing. Zhao et al. [271] introduced a dual crosslinked PAM-co-DAAM hydrogel system that retained 88% of its voltage output after healing, demonstrating its robustness for tactile sensing applications. These advancements highlight the growing importance of self-healing hydrogels in the development of multifunctional, transparent, and self-powered tactile sensors for next-generation wearable communication systems.

#### 5.3.2. Self-Healable Hydrogel-Based SH-TENG

**Figure 16:**
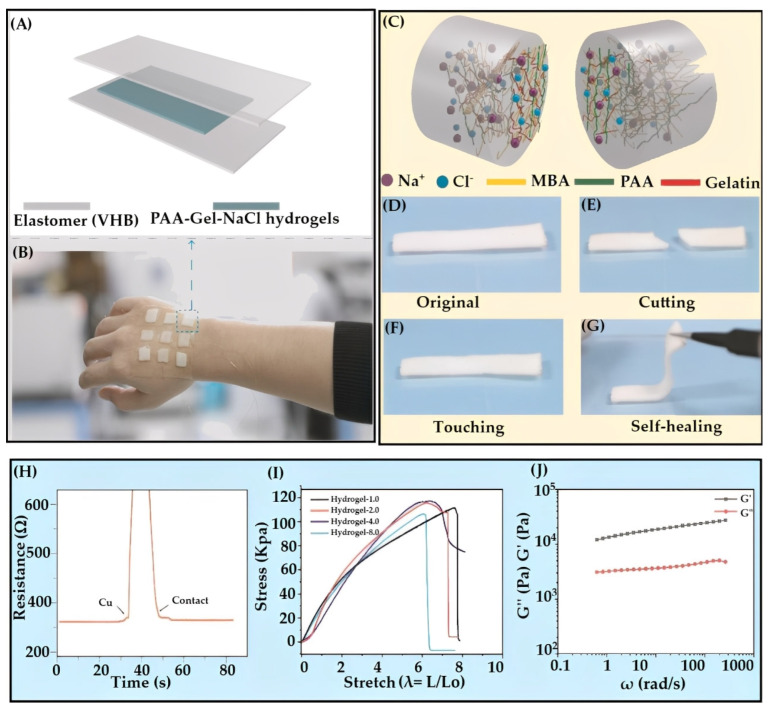
Design and performance of a self-healing triboelectric nanogenerator (SH-TENG) for electronic skin applications. (**A**) Schematic of the wearable SH-TENG model for electronic skin integration. (**B**) Optical image of a SH-TENG array patch (3 × 3 pixels) attached to the back of the hand. (**C**) Structural schematic of the PAA–Gel–NaCl ionic hydrogel. (**D**–**G**) Photographs showing the hydrogel before and after self-healing. (**H**) Demonstration of electrical recovery capability following mechanical damage. (**I**) Uniaxial tensile test results of PAA–Gel–NaCl hydrogels with varying gelatin content. (**J**) Storage and loss moduli of the hydrogel as a function of frequency under 1% strain. Adapted with permission from [272]. Copyright © 2021 MDPI.

Building on this concept, Han et al. developed a self-healing, stretchable hydrogel-based TENG (SH-TENG) using ion-conductive hydrogels (Figure 16A–J) [272]. The device achieved 800% elongation, self-healed within 2.5 min, and operated in a single-electrode mode with outputs of 22 V, 400 nA, and 2.9 μW/cm^2^. Its sensitivity to low-frequency human touch enabled applications in artificial skin and wearable tactile sensors, highlighting the potential of hydrogel networks for dynamic and resilient sensing platforms.

#### 5.3.3. Self-Healable and Ultra-Stretchable Gel-Based TENGs for High-Sensitivity Electronic Skins

**Figure 17:**
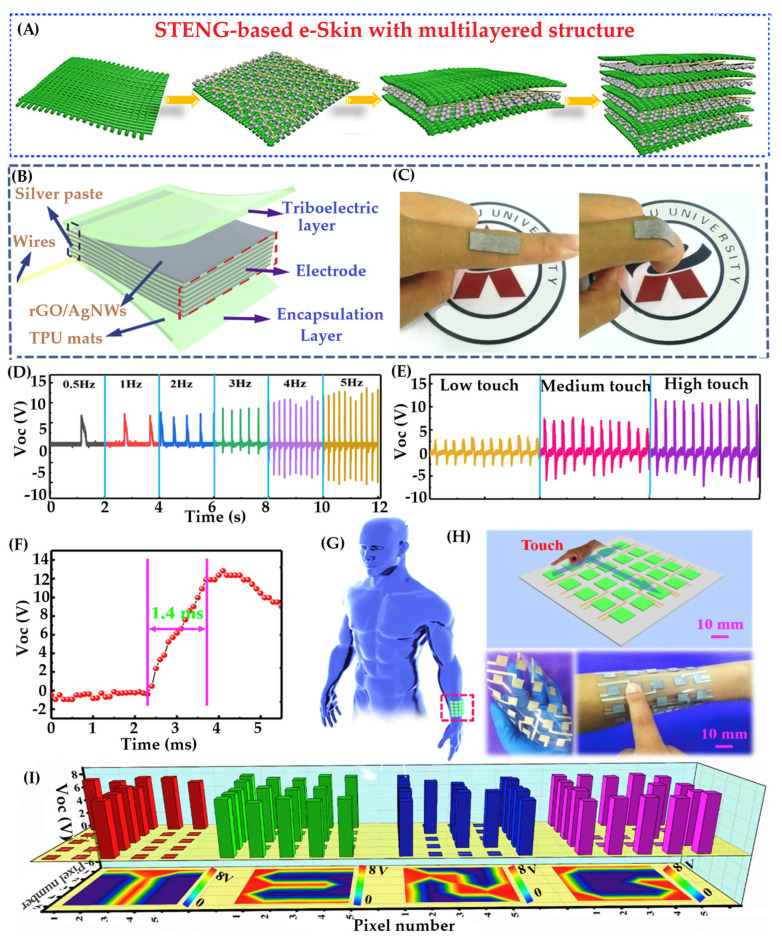
Applications of the self-powered hydrogel-based electronic skin (e-skin) for tactile sensing and energy harvesting. (**A**) Schematic illustration of the fabrication process, incorporating reduced rGO and silver nanowires (AgNWs) as conductive fillers. (**B**) Structural diagram of the stretchable triboelectric nanogenerator (STENG)-based e-skin. (**C**) Photograph of the e-skin conformally attached to a human finger. (**D**) Open-circuit voltage (VOC) responses of a single e-skin unit under varying frequencies and applied pressures. (**E**) Electrical output under finger tapping at different intensities. (**F**) Response time of the e-skin under a 5 Hz stimulus. (**G**) Photographs showing the e-skin array mounted on a forearm. (**H**) Schematic of a flexible tactile sensing array composed of 5 × 5 pixels. (**I**) Voltage mapping corresponding to the movement trajectory of the index finger across the sensing array. Adapted with permission from [273]. Copyright © 2020 Elsevier.

Gel-based TENGs have also been successfully applied in the development of ultra-flexible electronic skins for tactile sensing and energy harvesting. In a pioneering study, Zhou et al. developed a flexible and self-powered electronic skin (e-skin) utilizing an ultra-stretchable triboelectric nanogenerator (STENG) composed of multilayered thermoplastic polyurethane (TPU), silver nanowires (AgNWs), and reduced graphene oxide (rGO) (Figure 17A–I) [273]. The integration of highly stretchable TPU fibrous mats with a synergistic AgNWs/rGO microstructure enabled the e-skin to achieve excellent mechanical flexibility, sustaining up to 200% strain without performance degradation. The compact 2 × 2 cm^2^ device delivered a high open-circuit voltage of 202.4 V and a peak power density of 6 mW/m^2^, making it a promising candidate for sustainable energy harvesting. In addition to its energy generation capabilities, the e-skin exhibited high pressure sensitivity (78.4 kPa^−1^) and a rapid response time of 1.4 ms, allowing it to detect both the strength and trajectory of mechanical stimuli with precision. This work presents a practical approach for designing high-performance, self-powered e-skins suitable for applications in soft robotics, human–machine interfaces, and the Internet of Things.

Recent studies have further advanced the design of self-healing hydrogel-based TENGs for electronic skin applications. For example, Qin et al. developed a multifunctional TENG using a double-network hybrid hydrogel composed of polyacrylamide/poly(acrylic acid)/MXene/PEDOT:PET, which demonstrated excellent flexibility, self-healing ability, and stability. This system was capable of harvesting energy from human motion and monitoring dance postures, including facial expressions and joint movements [49].

Another notable example is the PTSM hydrogel-based TENG developed by Zhang et al., which utilized a composite of polypropylene amine, tannic acid, sodium alginate, and MXene. This hydrogel exhibited ultra-stretchability (>4600%), strong adhesion, and rapid self-healing. The resulting TENG was integrated into a glove-based human–machine interaction system capable of gesture visualization and object recognition with high accuracy [146].

These examples underscore the growing importance of self-healing hydrogels in developing multifunctional, self-powered electronic skins that combine energy harvesting, tactile sensing, and human–machine interaction capabilities for next-generation wearable technologies.

#### 5.3.4. Wearable TENGs for Health Monitoring

**Figure 18:**
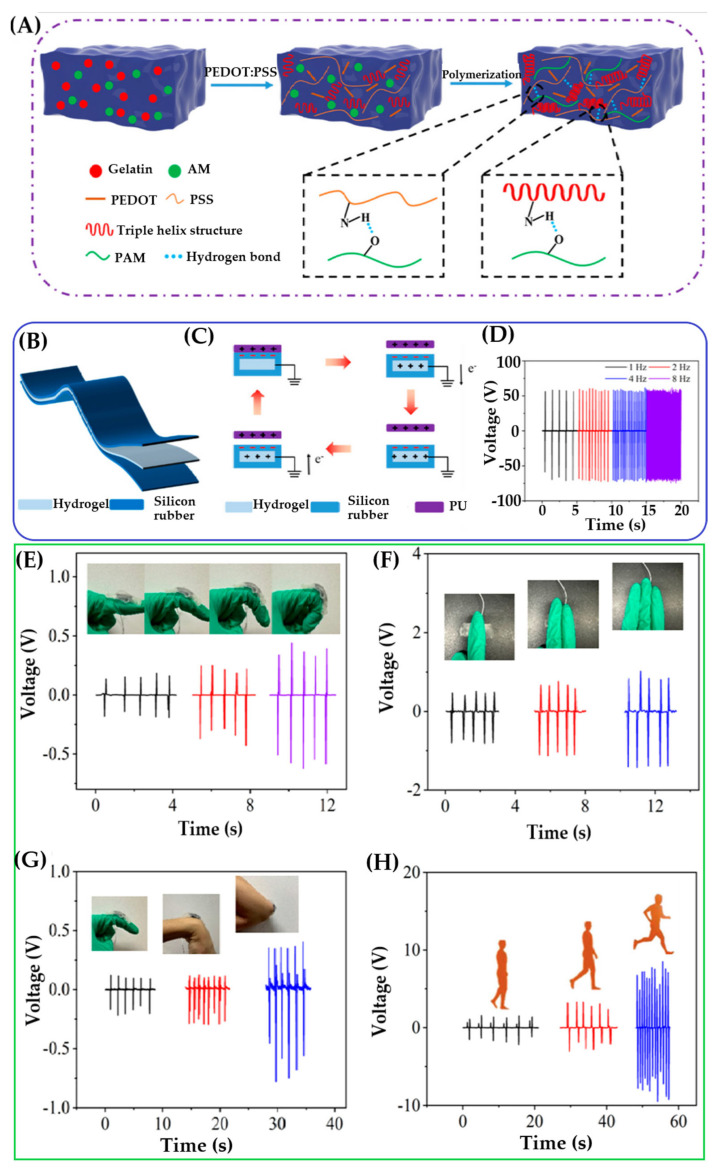
Design and sensing performance of a PGP-TENG based on a PAM/Gelatin/PSS:PEDOT hydrogel. (**A**) Schematic illustration of the synthesis process for the PAM/Gelatin/PSS:PEDOT (PGP) hydrogel. (**B**) Structural diagram of the PGP-based triboelectric nanogenerator (PGP-TENG). (**C**) Working mechanism of the PGP-TENG under contact-separation motion. (**D**) Output voltage response of the PGP-TENG at varying contact-separation frequencies. (**E**) Real-time tracking of finger movements using the PGP-TENG. (**F**) Detection of finger tapping events. (**G**) Monitoring of joint movements including fingers, wrists, and elbows. (**H**) Recognition of human locomotion states such as walking and running. Adapted with permission from [236]. Copyright © 2025 MDPI.

Wearable TENGs have emerged as promising tools for real-time health monitoring, driven by innovations in soft and responsive materials. Huang et al. advanced this field by engineering a PEDOT:PSS-doped gelatin-acrylamide hydrogel, resulting in a high-performance TENG with an output voltage of 192 V, a broad sensing range (0–1690%), and a rapid response time of 0.15 s (Figure 18A–H) [236]. This hydrogel-based TENG was successfully applied to monitor joint movements and gait patterns, demonstrating its utility in wearable health monitoring and human–machine interfaces.

In a more application-specific context, Yang et al. designed a self-healing poly(vinyl alcohol)-based hydrogel TENG sandwiched between silicone elastomer films. This device recovered its electrical performance within 10 min at room temperature and was used for muscle motion monitoring and photothermal therapy, showcasing its potential in rehabilitation and joint recovery [274].

In a more application-specific context, Zhang et al. developed an instant-healing hydrogel-based TENG using a PVA/HO-mCNT/tannic acid composite. This device achieved a high open-circuit voltage of 398 V and demonstrated excellent anti-freezing and water-retention properties. It was successfully used for handwriting recognition and non-contact sensing, highlighting its potential in intelligent human–machine interfaces and environmental monitoring [275].

Complementing these innovations, a comprehensive review by Li et al. emphasized the role of dynamic covalent polymers, supramolecular elastomers, and ion-conductive hydrogels in self-healing TENGs. These materials enable rapid damage recovery and stable electrical performance, making them ideal for artificial skin and wearable health monitoring applications [226].

Additionally, Zhang et al. reviewed the integration of stretchable hydrogels with high ionic conductivity and self-healing properties into on-skin electronics. Their work highlights strategies for optimizing structural design and enhancing user comfort, with applications in health monitoring, motion tracking, and human–machine interaction [276].

Together, these advancements underscore the transformative role of self-healing hydrogel-based TENGs in wearable health monitoring systems, offering robust mechanical resilience, high sensitivity, and energy autonomy for next-generation biomedical and human–machine interface technologies.

## 6. Challenges and Future Perspectives

Gel-based TENGs have emerged as promising candidates for flexible, self-powered sensing systems across biomedical, environmental, and wearable electronics domains. Despite their rapid development and demonstrated versatility, several critical challenges remain that must be addressed to enable their widespread deployment in real-world applications. These challenges span material stability, system integration, scalability, and the incorporation of emerging technologies.

### 6.1. Dehydration, Stability and Durability, and Integration Challenges of Gel-Based TENGs

One of the foremost limitations of gel-based TENGs is their vulnerability to environmental stressors such as dehydration, temperature fluctuations, and mechanical fatigue. In hydrogels, dehydration remains a major limitation, particularly for long-term wearable applications [277,278,279]. To address this, researchers are exploring anti-drying coatings, encapsulation strategies, and the use of organohydrogel composites that retain moisture under ambient conditions [280]. Integration with electronics also poses challenges due to the mismatch in mechanical properties and the need for stable electrical interfaces. Flexible interconnects, conductive adhesives, and hybrid packaging techniques are being developed to improve system-level integration [4,281].

Scalability is another critical issue, as many high-performance gels require complex synthesis or costly materials. Efforts are underway to develop printable gel inks and roll-to-roll fabrication methods to enable large-scale production [4,55,282]. Hydrogels, in particular, are susceptible to water loss, which can degrade their mechanical and electrical properties over time. While organogels and ionogels offer improved stability, long-term durability under cyclic loading and exposure to harsh conditions remains a concern. Lu et al. emphasized that enhancing the chemical robustness and environmental adaptability of gel matrices—through crosslinking strategies, hydrophobic modifications, and encapsulation—is essential for maintaining consistent performance in practical settings [34].

### 6.2. Integration with Electronics and Data Systems

For gel-based TENGs to transition from laboratory prototypes to functional devices, seamless integration with electronic components and data acquisition systems is imperative. This includes compatibility with flexible substrates, wireless communication modules, and low-power microcontrollers. However, the soft and deformable nature of gels poses challenges in establishing stable electrical interfaces and maintaining signal fidelity during dynamic motion. The development of conductive gel composites and hybrid architectures has shown promise in bridging this gap, but further work is needed to standardize fabrication protocols and ensure reliable performance across diverse operating conditions [34].

### 6.3. Scalability and Cost-Effectiveness

Scalability remains a significant bottleneck in the commercialization of gel-based TENGs. Many high-performance gels rely on complex synthesis routes or expensive nanomaterials, which hinder mass production. Simplified device architectures—such as single-layer gel electrodes and printable gel inks—have been proposed to reduce fabrication complexity and cost [40]. Nonetheless, achieving consistent quality, reproducibility, and affordability across large-scale manufacturing processes remains a challenge. Collaborative efforts between academia and industry are essential to develop scalable production methods and identify sustainable material sources.

### 6.4. Emerging Trends: Self-Healing Gels, Hybrid Systems, and AI-Assisted Sensing

To overcome existing limitations and expand the functionality of gel-based TENGs, several emerging trends are gaining momentum. Self-healing gels, capable of autonomously repairing mechanical damage, offer enhanced durability and longevity in wearable and implantable devices. Wang et al. reported hydrogel-based TENGs with spontaneous healing capabilities within one minute, without external stimuli, demonstrating their potential for long-term use in dynamic environments [40].

Hybrid systems that combine ionic and electronic conductivity, or integrate multiple sensing modalities (e.g., pressure, temperature, and chemical), are being developed to improve versatility and responsiveness. Moreover, hybrid architectures that incorporate triboelectric, piezoelectric, and thermoelectric mechanisms can broaden the range of detectable stimuli and enhance energy conversion efficiency [283,284,285,286,287].

Artificial intelligence (AI) and machine learning are also emerging as transformative tools in gel-based TENG applications [288]. Future research should focus on the convergence of these technologies to enable adaptive sensing, real-time diagnostics, and predictive analytics in wearable and environmental monitoring systems. Integrating AI with TENG-based sensors facilitates real-time data interpretation, adaptive control, and predictive diagnostics, paving the way for smart, autonomous sensing platforms tailored for personalized healthcare, environmental monitoring, and intelligent robotics [288,289,290,291].

In conclusion, while gel-based TENGs have made significant strides in flexible sensing, addressing challenges related to stability, integration, scalability, and multifunctionality is essential for their transition from experimental systems to practical technologies. Continued interdisciplinary research, coupled with advances in materials science, electronics, and data analytics, will shape the future landscape of gel-based energy harvesting and sensing systems [288,292,293].

## 7. Summary and Outlook

Polymer gel-based TENGs have emerged as versatile platforms for energy harvesting and sensing applications, owing to their inherent mechanical compliance, biocompatibility, and tunable conductivity. These properties have enabled the development of flexible, stretchable, and multifunctional devices suitable for diverse applications in health monitoring, environmental sensing, and tactile interfaces.

This review has highlighted the critical roles of gel architecture, conductivity mechanisms, and surface morphology in optimizing TENG performance. The integration of various gel types—including hydrogels, aerogels, ionic gels, and composite gels—has led to significant improvements in triboelectric output, sensitivity, and device resilience. The adaptability of gel-based systems across different working modes and material classifications underscores their potential in next-generation soft electronics.

Despite these advancements, several key challenges remain. Environmental stability, particularly dehydration in hydrogels, continues to limit long-term performance. Additionally, mechanical fatigue, signal reliability under dynamic conditions, and integration with electronic components pose barriers to real-world deployment. Scalability is another concern, as many high-performance gels require complex synthesis or costly materials.

To address these issues, emerging strategies such as anti-drying coatings, encapsulation, and organohydrogel composites are being explored. Furthermore, printable gel inks and roll-to-roll fabrication methods are under development to enable large-scale production.

Looking forward, self-healing gels capable of restoring mechanical and electrical properties after damage are expected to enhance device longevity and reliability. Hybrid systems that combine triboelectric, piezoelectric, and thermoelectric mechanisms can broaden the range of detectable stimuli and improve energy conversion efficiency. The integration of artificial intelligence (AI) and machine learning with gel-based TENGs opens new avenues for adaptive sensing, real-time diagnostics, and predictive analytics, particularly in wearable and environmental monitoring systems.

In conclusion, the future of gel-based TENGs lies in interdisciplinary collaboration across materials science, electronics, and data analytics. Innovations in gel chemistry, device engineering, and system integration will be essential to fully realize the potential of these materials in sustainable, intelligent, and adaptive sensing technologies.

## Figures and Tables

**Table 1:** Gel-based TENGs classified by network structure.

Type	Material Composition	Key Performance	Voc	Isc	Ref.
Single-Network Gel	PAM	Enhanced interface tear resistance by 1200%	311 V	32.4 μA	[97]
Single-Network Gel	PAM	High optical transmittance (89%) and stable performance across −25 °C to 60 °C	120 V	16.6 μA	[98]
Single-Network Gel	ACMO	Achieved instantaneous power density of 40 mW/m^2^	40 V	138 μA	[99]
Single-Network Gel	PVA	High transmittance (92%) and uniaxial stretchability up to 700%	50 V	13 μA	[100]
Double-Network Gel	PVA/PAAm	High bacteriostasis and biocompatibility	270.5 V	16.2 μA	[106]
Double-Network Gel	PHEA-co-AA	Pressure detection limit of 0.016 kPa	182 V	2.61 μA	[102]
Double-Network Gel	PAA/rGO	Stretchability up to 650%	21.77 V	48.7 μA	[22]
Multi-Network Gel	MCC	High sensitivity with 60 ms response time	10.3 V	—	[107]
Multi-Network Gel	CMC	Stable performance at −40 °C	70 V	—	[105]

N.B: PAM: polyacrylamide; ACMO: acrylamide-2-methylpropane sulfonic acid; PVA: polyvinyl alcohol; PAAm: polyacrylamide; PHEA: poly(N-hydroxyl acrylamide); AA: acrylic acid; rGO: reduced graphene oxide; MCC: microcrystalline cellulose; and CMC: carboxymethyl cellulose.

**Table 2:** Gel-based TENGs classified by matrix material.

Matrix Type	Material Composition	Key Performance	Voc	Isc	Ref.
Inorganic Gel	Succinonitrile/Acrylic Ester UV Glue	Reduced VO_2_ infrared transmittance by 28%	8 V	—	[113]
Inorganic Gel	AMPS/PEGDA	High stretchability (2750%), conductivity (1.375 S/m)	135 V	—	[116]
Organic Gel	Chitosan/Catechol	Biocompatible hydrogel for wearable applications	110 V	3.8 μA	[128]
Organic Gel	Hyaluronic Acid (HA)	Biocompatible and biodegradable membrane	20 V	—	[120]
Organic Gel	PVC with Dioctyl Glyoxylate (DOA)	High durability over 30 days of cyclic testing	198 V	—	[123]
Organic Gel	AAm/THMA	Tensile strength: 0.25 MPa; fracture strain: 1015%	187 V	6 μA	[124]
Composite Gel	Au/Ecoflex	Stretchability: 900%; energy conversion efficiency: 26%; self-healing	375 V	4 μA	[129]
Composite Gel	PVA/g-C_3_N_4_	Cost-effective and recyclable hydrogel electrode	45 V	0.8 μA	[130]

N.B: AMPS: 2-acrylamido-2-methylpropane sulfonic acid; PEGDA: polyethylene glycol diacrylate terminated; THMA: N-[tri(hydroxymethyl)methyl]acrylamide; and g-C_3_N_4_: graphitic carbon nitride.

**Table 3:** Gel-based TENGs classified by dielectric material.

Dielectric Type	Material Composition	Key Performance	Voc	Isc	Ref.
Hydrogels	MXene/PVA	Stretchability up to 200%	230 V	—	[164]
Hydrogels	PVA/PEG/MXene	High surface charge density	212 V	—	[145]
Hydrogels	PAM/TA/SA/AM/MXene	Strain coefficient of 6.6	190 V	1.25 μA	[165]
Hydrogels	PAM/BP	Stretchability exceeding 1000%	130 V	2 μA	[147]
Hydrogels	Gelatin/Silver Nanoparticles	Stretchability over 2600%; antibacterial functionality	225 V	1.5 μA	[148]
Aerogels	Silk Fibroin (SF)	Biocompatibility, lightweight, and bacteriostatic effect	545 V	16.3 μA	[126]
Aerogels	PDA/Ag/CNF	Antibacterial activity >99%	211 V	18.1 μA	[166]
Ionogel	NH_2_-HBP—Nano SiO_2_ and Silicone Rubber	Stretchable (812%), thermally stable (−80 to 250 °C), washable, self-powered sensing	247 V	11.7 μA	[159]
Ionogel	PVA-H_3_PO_4_	Stretchable (1058%), transparent, EDL-enhanced output, no dehydration	992 V	44.8 μA	[160]
Ionic Gels	EMIM-TFSI	Flame-retardant properties	150 V	45 μA	[161]
Ionic Gels	EMIM-TFSI	High thermal stability	122 V	12 μA	[162]
Ionic Gels	EMIM-TFSI/MXene	Wide pressure response range (0.6 Pa to 1 MPa)	180 V	—	[163]

N.B: TA: tannic acid; SA: sodium alginate; AM: acrylamide; BP: black phosphorus; PDA: polydopamine; CNF: cellulose nanofibers; and EMIM-TFSI: 1-ethyl-3-methylimidazolium bis(trifluoromethanesulfonyl)imide.

**Table 4:** Classification of gel materials in TENGs with advantages, disadvantages, and references.

Gel Type	Classification	Roles in TENG	Advantages	Disadvantages	Ref.
Single-network Gel	Network Structure	Electrode, Tribolayer	Simple synthesis, tunable mechanical properties	Limited toughness and durability	[95]
Double-network Gel	Network Structure	Electrode	Enhanced mechanical strength, toughness, and stretchability	More complex synthesis, potential phase separation	[25,95]
Multi-network Gel	Network Structure	Electrode	Superior mechanical resilience, multifunctionality	High fabrication complexity and cost	[104]
Inorganic Gel	Matrix Material	Tribolayer	High thermal stability, chemical resistance	Poor flexibility, limited biocompatibility	[108,109,110]
Organic Gel	Matrix Material	Electrode, Tribolayer	Biocompatibility, flexibility, tunable conductivity	Lower mechanical strength, sensitive to environment	[133,167,168]
Composite Gel	Matrix Material	Electrode, Tribolayer	Synergistic properties, improved conductivity and strength	Complex fabrication, cost-intensive	[79,169,170]
Hydrogel	Contained Liquid Phase	Electrode, Tribolayer, Substrate	High water content, biocompatibility, ionic conductivity	Prone to dehydration, poor long-term stability	[25,38,143,171,172,173,174]
Aerogel	Contained Liquid Phase	Tribolayer	Lightweight, porous, thermal insulation	Brittle, limited strain sensing range	[153,174,175,176]
Ionic Gel	Contained Liquid Phase	Electrode	High ionic conductivity, stable under various conditions	Potential leakage, limited mechanical strength	[29,99,177,178]

## Data Availability

No new data were created or analyzed in this study.
